# Investigating adaptation to environmental variability in forest trees through molecular phylogenetic analysis

**DOI:** 10.1371/journal.pone.0338893

**Published:** 2025-12-23

**Authors:** Cesare Garosi, Cristina Vettori, Roberta Ferrante, Donatella Paffetti

**Affiliations:** 1 Department of Agriculture, Food, Environment and Forestry (DAGRI), University of Florence, Piazzale delle Cascine 18, Florence, Italy; 2 Institute of Bioscience and Bioresources (IBBR), Division of Florence, National Research Council of Italy (CNR), Via Madonna del Piano 10, Sesto Fiorentino, Italy; 3 NBFC, National Biodiversity Future Center, Palermo, Italy; Stockholm University, SWEDEN

## Abstract

We conducted a molecular phylogenetic analysis of the abiotic stress response in 13 key European forest species (*Fagus sylvatica* L., *Quercus robur* L., *Quercus ilex* L., *Quercus pubescens* Willd., *Quercus suber* L., *Quercus lobata* L., *Juglans regia* L., *Populus trichocarpa* L., *Pinus taeda* L., *Pinus nigra* J.F. Arnold, *Pinus pinea* L., *Pinus pinaster* Aiton and *Abies alba* Mill.) to clarify how different abiotic stressors have influenced their adaptation. The study on the evolution of abiotic stress responses in these species, seeks to uncover the factors driving their distinct evolutionary pathways of adaptation. We created the dataset by collecting data from genomic dataset on genes relevant to the response to abiotic stress in the target species dataset. Then, we used the data in the dataset to search for possible orthologs in the studied species dataset. A matrix was created with sequences of each identified ortho-group, closely related to the analyzed genes, and phylogenetic relationships were reconstructed using the maximum likelihood (ML) method. Pairwise estimates of synonymous and nonsynonymous substitutions per site (Ks and Ka, respectively) were calculated using the ML method. Analysis of 616 genes associated with abiotic stress response revealed 347 genes in angiosperms species, with *F. sylvatica* having the highest count, and 269 genes in conifers, where *A. alba* contributing the most. Drought stress exhibited the highest number of shared genes, while freezing stress showed the least. Substitution rate analysis indicated higher average values in angiosperms species, with a stronger signature of adaptive evolution in conifers, as suggested by the higher Ka/Ks ratio. The study unveils distinctive patterns in the evolutionary dynamics of molecular responses to abiotic stresses between the 13 key forest tree species. Lower substitution rates in conifers suggest unique constraints, likely influenced by larger genomes and ancient lineage divergence. The prevalence of Ka/Ks values below unity emphasizes strong selective constraints, highlighting the conservation of abiotic stress response mechanisms across diverse lineages.

## 1 Introduction

Forest trees have faced environmental stresses throughout their 385 million years of evolution, significantly influencing their adaptation, diversification, and extinction [1–4]. The ability of these species to adapt to various abiotic stresses—such as temperature, drought, salinity, flooding, light quantity, and atmospheric CO_2_ concentration—has been central to their survival and evolution [5–7]. Understanding the genetic basis of these adaptive responses is crucial, as it reveals how evolution is shaped by the presence of adaptive alleles with varying effects [6,8]. Observations on the number, frequency, effect size, and genomic distribution of alleles associated with adaptation must be interpreted within the context of evolutionary processes [6,8]. These aspects, constituting a trait’s genetic architecture, significantly influence evolutionary outcomes and provide insights into the mechanisms of evolution.

The responses of higher plants to abiotic stresses have been extensively studied at both physiological and ecological levels in controlled and field conditions [9–17]. The high plasticity potential of trees enables them to mitigate and adapt to chronic stresses and extreme events [18]. A multidisciplinary approach is essential for understanding forest ecosystem adaptation in response to climate change, emphasizing the importance of implementing genetic analysis [19]. Genetic and genomic studies have explored how trees respond to stress, demonstrating that forest species typically exhibit large effective population sizes, moderate to high levels of genetic diversity, and frequent occurrences of locally adapted ecotypes [20–25]. Despite these characteristics, rates of molecular evolution across species tend to be slow [26]. Additionally, genome sizes vary among tree species, ranging from 0.4 Gbp to 31 Gbp [5]. Angiosperms genomes are better understood than conifer genomes due to the large size and high repetitive element content of the latter, which complicates assembly and bioinformatics [10,27]. Sequencing efforts in gymnosperms, which possess the largest tree genomes, reveal that much of the variation in genome size results from transposable element dynamics and gene family evolution [28–32]. For example, duplication events of specific gene families may enhance the ability of the trees to colonize marginal habitats [27]. Comparative studies suggest that the evolution of angiosperms was shaped by whole-genome duplication (WGD) events, resulting in higher rates of speciation [33,34]. In contrast, gymnosperm genomes exhibit lower flexibility and evolutionary dynamics, characterized by fewer WGD events, sparse chromosomal rearrangements, and slow mutation rates, which contribute to reduced levels of genomic structural variation and speciation rates [19,26–29,33–35]. In addition to significant variations in non-coding regions, comparative studies indicate that protein-coding genes and gene families show notable sequence similarities—approximately 60% between conifers and angiosperms species and around 80% within conifers [36]. These functional differences arise from variations in nucleotide substitution rates and the formation of copy number variants [35–38].

The extensive gene flow and large geographical ranges of trees enable researchers to differentiate between neutral and selective evolutionary processes [32]. Their longevity and heterogeneous geographical distributions make trees ideal for addressing key evolutionary questions concerning historical climatic fluctuations and adaptation involving shifts in allele frequencies [32,39–41]. The adaptation to the local environment can favor specific genetic architecture, where large-effect alleles are clustered within fewer genomic regions (candidate genes), in contrast to expectations under global adaptation [8].

In natural populations, migration, pleiotropy, mutation rate, and temporal environmental variability can act as limiting factors and constrain the evolution of such architectures. In population genetic models, adaptive evolution is explained as changes in allele frequencies at individual loci under selection. Strong selection at a single locus generally results in substantial shifts in allele frequency during adaptation (hard-sweep model) [42–44]. In contrast, the quantitative genetics framework suggests that numerous alleles contribute small, interchangeable effects to traits, enabling large changes in traits to occur through relatively modest shifts in allele frequencies across many loci (partial, selective, and soft-sweep model) [6–8,42–44]. Estimates indicate that a significant proportion of non-synonymous substitutions are fixed by selection, demonstrating that selection plays a crucial role in the long-term evolution of genomic sequences [45–47]. However, much adaptive variation is also guided by alleles of small effect, with adaptation from standing genetic variation being a common evolutionary process [8,48–50].

Many of the large-effect adaptive alleles identified to date predominantly drive local adaptation, rather than global adaptation, or are maintained by balancing selection [6–8]. Local adaptation patterns are believed to involve fewer higher-impact alleles and more closely related, than global adaptation. The process of adaptation is central to evolution, and many fundamental questions are directed toward understanding how local and global adaptation have shaped the evolution of forest species [8]. An interesting perspective on the evolution of adaptation in higher plants is that the molecular response to abiotic and biotic stresses appears to be highly conserved and relatively easy to evolve, with numerous origins within angiosperms and gymnosperm lineages. [7,12,51,52]. They promote the use of macroevolutionary models as a robust approach to analyzing the evolution of abiotic stress responses [7]. These analyses often integrate detailed knowledge of individual species with broader insights across multiple species, providing a richer understanding of evolutionary history.

Despite numerous studies of comparative genomics, phylogeny, and evolution of forest species, there is a lack of information regarding genes related to responses to abiotic stresses. The study of genes associated with adaptation in forest species is essential for understanding the evolutionary history of these organisms. [7–12,52].

In our study, we used a dataset of 616 genes associated with responses to abiotic stressors (drought, heat, salt, frost, and cold). This dataset was derived from genomic and transcriptomic data of 13 forest tree species, which include eight broadleaves: *Fagus sylvatica* L., *Quercus robur* L., *Quercus ilex* L., *Quercus pubescens* Willd., *Quercus suber* L., *Quercus lobata* L., *Juglans regia* L., and *Populus trichocarpa* L.; and five conifer species: *Pinus taeda* L., *Pinus nigra* J.F. Arnold, *Pinus pinea* L., *Pinus pinaster* L., and *Abies alba* Mill. This dataset was used to carried out a phylogeny analysis and investigate the different evolutionary histories to better understand the causes of divergence related to genes associated with responses to abiotic stressors. The aim is to understand the influence of local and global environment adaptation on the events that characterized the evolutionary history of these species.

## 2 Materials and methods

**Fig 1:**
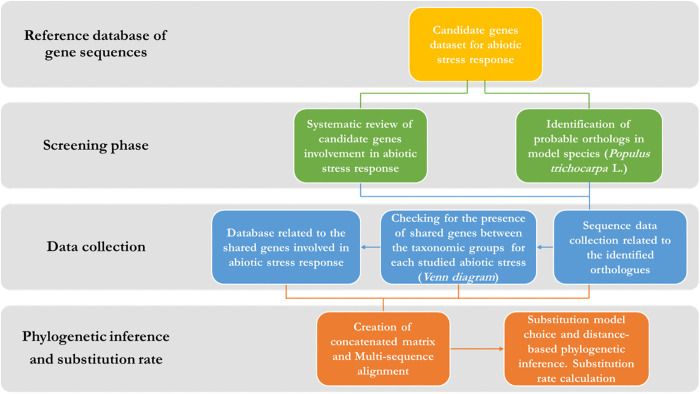
Experimental design: flowchart of analytic phases followed to perform the analysis.

The experimental design carried out for the analysis is illustrated in Fig 1. The design is divided into four main phases, which serve as a schematic framework to clarify the analytical process. The initial phase involves the development of a starting dataset, which includes identifying and selecting genes relevant to responses to one or more abiotic stresses in the target species, 8 broadleaf species (*Fagus sylvatica* L., *Quercus robur* L., *Quercus ilex* L. and *Quercus pubescens* Willd., *Quercus suber* L., *Quercus lobata* L., *Juglans regia* L., and, *Populus trichocarpa* L.) and 5 conifers species (*Pinus taeda* L., *Pinus nigra* J.F. Arnold., *Pinus pinea* L., *Pinus pinaster* L., and *Abies alba* Mill.). The second and third phases are essential for preparing data for comparative analysis. These phases involve screening gene sequences to identify orthologs in model species and addressing potential biases associated with abiotic stresses correlated with the studied genes. The final phase comprises phylogenetic and evolutionary dynamics (substitution rates) analysis using the obtained sequences. Each phase will be discussed in detail in the subsequent sections dataset.

### 2.1 Reference database of gene sequences

Available literature regarding molecular response to abiotic stresses (drought, cold, frost, salinity, heat stress) for each species was collected using the most relevant datasets as described in the section systematic review. For species names, both the Latin binomial and common names (i.e., *Fagus sylvatica* L. and/or beech) were used. The identified papers were analysed to derive sequence ID corresponding to the reported candidate genes. These IDs were used to retrive FASTA files containing gene sequences for the candidate genes, from the principal genomic datasets (NCBI, EnsemblPlant, Panther, PlantGDB, and Treegenes). The last step, before the development of the dataset, involved sequences validation and annotation to minimize the possibility of any errors during the next steps. Sequences were validated through alignment with similar sequences using the BLAST algorithm [53]. Alignment was performed using the MAFFT v. 7 and MEGAX software tools [54,55]. By aligning complete sequences, including coding DNA sequences (CDS) and complementary DNA (cDNA), we reconstructed the gene sequences. An *ad hoc* dataset was then created to consolidate all the information on each candidate gene (S1 Table) dataset.

### 2.2 Screening phase

The screening phase involves a preliminary analysis of gene sequences obtained from the literature. This phase consists of two primary objectives: i) conducting a bibliographic search to determine whether the identified genes have been studied in relation to responses to other abiotic stresses, and ii) identifying potential orthologs in the target species.

#### 2.2.1 Systematic review.

We conducted a qualitative systematic review of candidate genes, listed in our reference dataset, in various abiotic stresses as stated in Garosi *et al*. (2022) [56]. We sought to determine whether the candidate genes identified in the initial phase of our analysis are also implicated in responses to abiotic stresses beyond those already documented in the reference dataset. The abiotic stresses under consideration are drought, heat, cold, salinity, and freezing dataset. To this end, we performed a literature search for each selected gene through major datasets (Scopus, Research Gate, Spring link, Web of Science) and search engines (PubMed and Google Scholar) using the following search terms: “gene ID” + “plant*” + “abiotic stress” + “response*” (S2 Table). We did not use any inclusion criteria for this systematic review, as the objective of this step was to observe if the genes had been studied in response to different abiotic stresses beyond those identified and annotated in the reference dataset. To reduce potential bias, we consulted multiple sources (Scopus, Web of Science, PubMed, ResearchGate, SpringerLink, Google Scholar) and considered evidence from different approaches, including RNA-seq data, transcriptomic analyses, and environmental association studies.

#### 2.2.2 Ortholog identification.

The identification of orthologs was done as reported by Horiike *et al*. [57] and Ran *et al*. [58]. Gene sequences for the sampled taxa were derived using the BLAST algorithm (blastn algorithm with the default parameters and the standard core nucleotide database – core_nt) and various datasets (GenBank, NCBI, Phytozome). We used MEGAX and MAFFT v.7 alignment software to verify the imported sequences and align them with the gene sequences available in the reference dataset. Successively, we used Ortholog-finder to define the ortho-groups, which were further filtered based on the following criteria: (i) each species has at least one sequence; (ii) at least 5 species are present; and (iii) at least 2 species are present for each phylum [58]. The ortho-groups obtained were used to construct preliminary NJ trees using MAFFT v. 7 [54], with the GTR-GAMMA model. Redundant pairs and paralogs were identified and removed based on the tree topology of each orthogroup.

Moreover, we used the OrthoFinder v2.5.5 algorithm [57] to identify possible orthologs between species. Sequence similarity searches were performed with DIAMOND v2.1.x in -very-sensitive mode, applying the following filters: e-value ≤ 1e-5, minimum identity ≥ 30%, and alignment coverage ≥ 50% on both query and subject. Orthogroups were inferred using the MCL algorithm with an inflation parameter (I) = 1.5. The species tree was automatically inferred by OrthoFinder from gene trees using STAG and rooted with STRIDE; no external tree was imposed. Reciprocal best-hit (RBH) checks were not applied, since OrthoFinder assigns orthologs through a tree-based phylogenetic approach that minimizes paralogy more robustly than RBH alone. We then used profile-based alignment methods (MAFFT v.7) that consider domains and motifs conserved within gene families [54].

### 2.3 Data collection

The data collection consisted of identifying candidate genes present in both angiosperms and coniferous trees. Using a comparative genomics approach, we identified genes shared between broadleaf and conifer species and established a new reference dataset.

#### 2.3.1 Shared gene identification between angiosperms species and conifers.

Starting with the initial reference dataset, which was refined through ortholog identification and systematic searches, we performed a comparative analysis to determine which candidate genes were shared between broadleaf and conifer species dataset. We visualized the data in the dataset using a Venn diagram, obtained with the R package Venn Diagram [59]. The Venn diagram compares gene lists from a series of experiments and identifies genes shared between experiments or unique to an experiment in relation to stress. Complementarity in the molecular response to abiotic stresses between angiosperms and coniferous trees was observed both by considering the entire set of candidate genes and by considering genes involved in the response to individual stresses. After identifying gene sequences shared between the 13 studied forest species, we proceeded to create a second reference dataset (S2 Table), within which we reported all observed genes, relationship to abiotic stresses, sequence IDs obtained from GeneBank, and sequence IDs for the selected reference species (*Populus trichocarpa* L. and *Abies alba* Mill.). Despite the presence of the sequenced genome of *Picea abies* L. [30] (used as a model species for the study of conifer evolution), here we used *Abies alba* Mill. as the reference species [60]. We selected the reference species based on their ecological importance, the genome data availability and their relevance for genetic and genomics studies. We then created a matrix consisting of all the sequences of each identified ortho-group closely related to the genes found in common between angiosperms and coniferous trees during the comparative analysis.

### 2.4 Phylogenetic inference and substitution rate

The results of the previous two steps (Screening phase and Data collection) were used to carry out the phylogenetic inference and evolutionary divergence analyses. This phase consists in studying the sequences of protein-coding genes to assess the evolutionary history of the molecular response to abiotic stress in the target forest tree species.

#### 2.4.1 Gene concordance analysis.

To assess the congruence between individual gene trees and the concatenated phylogenetic tree, each gene alignment was first used to reconstruct a phylogenetic tree. Alignments were generated using MAFFT v7 [54], and initial phylogenetic reconstructions were performed in MEGAX [55] to visually inspect the topology of each gene tree. These analyses indicated that the topology of individual gene trees was broadly consistent with the concatenated species tree, justifying the inclusion of all loci in the concatenated analysis.

The full set of orthologous loci was concatenated into a supermatrix, and maximum likelihood (ML) phylogenetic reconstruction was performed using IQ-TREE2 [61]. The best-fit substitution model for each gene was automatically selected using the ModelFinder function (*-m MFP*), and branch support was evaluated with ultrafast bootstrap replicates (*-bb 100000*). To formally quantify gene-tree discordance, gene concordance factors (gCF) and site concordance factors (sCF) were calculated for each locus using IQ-TREE2, providing branch-specific measures of concordance and discordance..

#### 2.4.2 Concatenated matrix, multi-sequence alignment and phylogenetic analysis.

We analyzed the concatenated matrix of orthogroups to infer phylogenetic relationships underlying molecular responses to abiotic stress among the target species. All ortho-groups alignments were concatenated directly using R packages phangorn [62], ape [63] and apex [64]. Phylogenetic relationships were reconstructed using the partitioned and unpartitioned maximum likelihood (ML) method. The partitioned method consisted of treating each gene as a single partition and then grouping to analyse the results as a cluster. In contrast, the non-partitioned method consisted of analysing the matrix as a single unit, refined by a partitioning scheme in which the positions of the individual genes that comprised it were indicated. ML analyses were performed using various R packages [62–64]. For unpartitioned analyses, the GTR + G(4) + I model (General time reversible + Gamma distribution) was used, chosen using the ModelTest function of the R package Phangorn. For the unpartitioned analyses, node support was evaluated using 1,000,000 fast bootstrap replications. For the partitioned analyses, we used the same model as for the non-partitioned analyses. Node support for the partitioned analyses was also evaluated by 1,000,000 fast bootstrap replications. The tree was rooted using the root.tree function (ape package [63]), using the complete genome of *Ginkgo biloba* L. as outgroup [65]. Finally, using the Shimodaira-Hasegawa test (SH-test), Akaike Information Criterion (AICc), and Bayesian Information Criterion (BIC), we evaluated the confidence of the obtained trees (S3 and S4 Tables) [38].

#### 2.4.3 Substitution rate calculation.

Multiple sequence alignments for each of the shared genes were divided into two groups: one containing the eight angiosperms species and the other containing the five conifer species. This grouping facilitated further comparisons between these taxa. Alignment gaps and low-quality regions were manually removed. Pairwise estimates of synonymous substitutions per site (Ks), nonsynonymous substitutions per site (Ka), and the nonsynonymous/synonymous substitution rate ratio (ω or Ka/Ks, also known as *omega*) were calculated for species on terminal branches within each taxonomic group (angiosperms species and conifers). These calculations were performed using the maximum likelihood method of Goldman and Yang (1994), implemented in the codeml program from the PAML package (version 4.8) [65–67]. We used the codeml program, adjusting the following parameters: runmode = −2; seqtype = 1, model = 0, nsITES = 0 [26,68]. The analysis was repeated three times for each gene. For each sequence pair, only the results with the higher *ln*L (log likelihood) were retained. Following the methods applied by Buschiazzo *et al*. [68], we have checked for outlier identification. For conifers, we discarded 57 orthologues in total (45 with Ks > 2.5 and 12 with Ka > 1.5), and for angiosperms species, we discarded 39 genes (26 genes with Ks > 8, 4 genes with Ka > 1.5, and 26 genes with Ka/Ks ratio < 0.09). Thresholds were determined following empirical evidence, by plotting Ka as a function of Ks and excluding outliers from the main distribution. The final orthologue dataset contained 264 genes (123 conifers genes and 141 angiosperms species genes). 95% confidence intervals (CI) for evolutionary estimates were calculated based on 1,000 bootstrap replicates using R package bootES [69]. We discarded genes with Ks values lower than 0.01, as these values may result in inaccurate estimates of ω (Ka/Ks) [68] (S5 and S6 Table).

## 3 Results

### 3.1 Gene sequences data

Analysing a set of 616 genes associated with responses to abiotic stresses (drought, heat, salt, frost, and cold) we obtained 347 gene sequences from angiosperms species and 269 from conifers, all relevant to specific abiotic stresses (Table 1). For each sequence, we recorded the corresponding dataset ID (NCBI), the associated abiotic stress, the reference KEGG pathway, the results from the BLAST search, and the related bibliographic referencesdataset. The table is available in supplementary information (S1 Table).

**Table 1:** Number of genes observed for each species analysed.

Species	Number of genes^a^	Drought^b^	Heat^c^	Salt^d^	Frost^e^	Cold^f^
***Fagus sylvatica* L.**	183	163	34	10	25	25
***Quercus pubescens* Willd.**	28	28	28	28	28	28
***Quercus robur* L.**	119	62	75	6	65	65
***Quercus ilex* L.**	17	17	17	0	0	0
**Total (Angiosperms)**	347	270	154	44	118	118
***Abies alba* Mill.**	196	149	115	24	77	85
***Pinus pinea* L.**	28	27	2	2	3	2
***Pinus pinaster* Aiton**	32	21	12	12	12	12
***Pinus nigra* J.F.Arnold**	13	11	2	3	2	2
**Total (Conifers)**	269	208	131	41	94	101

^a^Report the total number (N) of genes for each species.

^b-f^The number of genes for each response to specific abiotic stress.

### 3.2 Shared gene identification and ortholog prediction

To mitigate potential biases in the analysis due to limited information on the involvement of the selected genes in abiotic stress responses, we conducted a systematic review [56]. As previously done in Garosi *et al*. [56], we have collected data regarding genes that are involved in the response to one or more abiotic stress. In published work, we had already obtained information regarding 24 candidate genes for abiotic stress response in *Fagus sylvatica* L. [56]. Nowadays, the literature search enabled us to collect papers (540) concerning the involvement of genes in the response to one or more abiotic stresses, and most genes are involved in the response to multiple abiotic stresses (Table 2).

**Table 2:** Number of genes observed for each species analysed after the results of systematic review.

Species	N° of genes^(a)^	Drought^(b)^	Heat^(c)^	Salt^(d)^	Frost^(e)^	Cold^(f)^	Shared^(g)^
***Fagus sylvatica*** **L.**	183	175	109	122	39	111	15
***Quercus pubescens* Willd.**	28	28	28	28	28	28	3
***Quercus robur* L.**	119	100	88	67	71	91	11
***Quercus ilex* L.**	17	17	17	11	1	7	0
**Total (Angiosperms)**	347	320	242	228	139	237	
***Abies alba* Mill.**	196	177	158	135	88	143	45
***Pinus pinea* L.**	28	28	10	15	5	13	1
***Pinus pinaster* Aiton**	32	31	22	26	15	25	2
***Pinus nigra* J.F. Arnold**	13	13	7	9	5	9	1
**Total (Conifers)**	269	249	197	175	113	190	

^a^Report the total number (N) of genes for each species.

^b-f^The number of genes for each response to specific abiotic stress.

^g^The number of genes involved in multiple stress response.

The systematic review enabled us to develop a more comprehensive dataset detailing the involvement of candidate genes in abiotic stress responses and adaptation. Following this, we identified potential shared genes among the selected target species. The automated identification of homologous genes across species has become increasingly crucial for functional annotation and evolutionary genomics [5,70]. Errors in ortholog prediction can significantly affect downstream analyses; consequently, there has been growing interest in high-quality ortholog prediction techniques [5,38,70]. Phylogenetic inference should always be based on related sequences, specifically, whose common ancestor is diverged as a result of speciation (orthologs), rather than a duplication event (paralogs) [38]. Young *et al*. [38], divide orthologs identification methodologies into two macro-categories: i) similarity-based methods and ii) phylogeny-based methods [38,71,72]. Comparative studies have shown that phylogeny-based methods offer greater specificity in ortholog identification [71–73].

To explore the complementarity of molecular responses to abiotic stresses, we analyzed the candidate genes—grouped by abiotic stress—across the target species, which were categorized into two major groups: conifers and angiosperms trees. The results revealed several candidate genes common to both broadleaf and conifer species in response to abiotic stresses. Notably, the highest number of shared genes was observed for drought stress response, while the lowest number was found for freezing stressdataset (Table 3).

**Table 3:** Number of genes for conifers and angiosperms trees divided according to their response to a specific abiotic stress. The last column shows the number of genes shared between broadleaves and conifers, subdivided according to their response to a specific abiotic stress.

Abiotic stress	N° of genes reported for conifers	N° of genes reported for broadleaves	N° of shared genes
**Drought**	269	320	264
**Salt**	175	228	166
**Cold**	190	237	184
**Heat**	197	242	185
**Frost**	113	139	111

**Fig 2:**
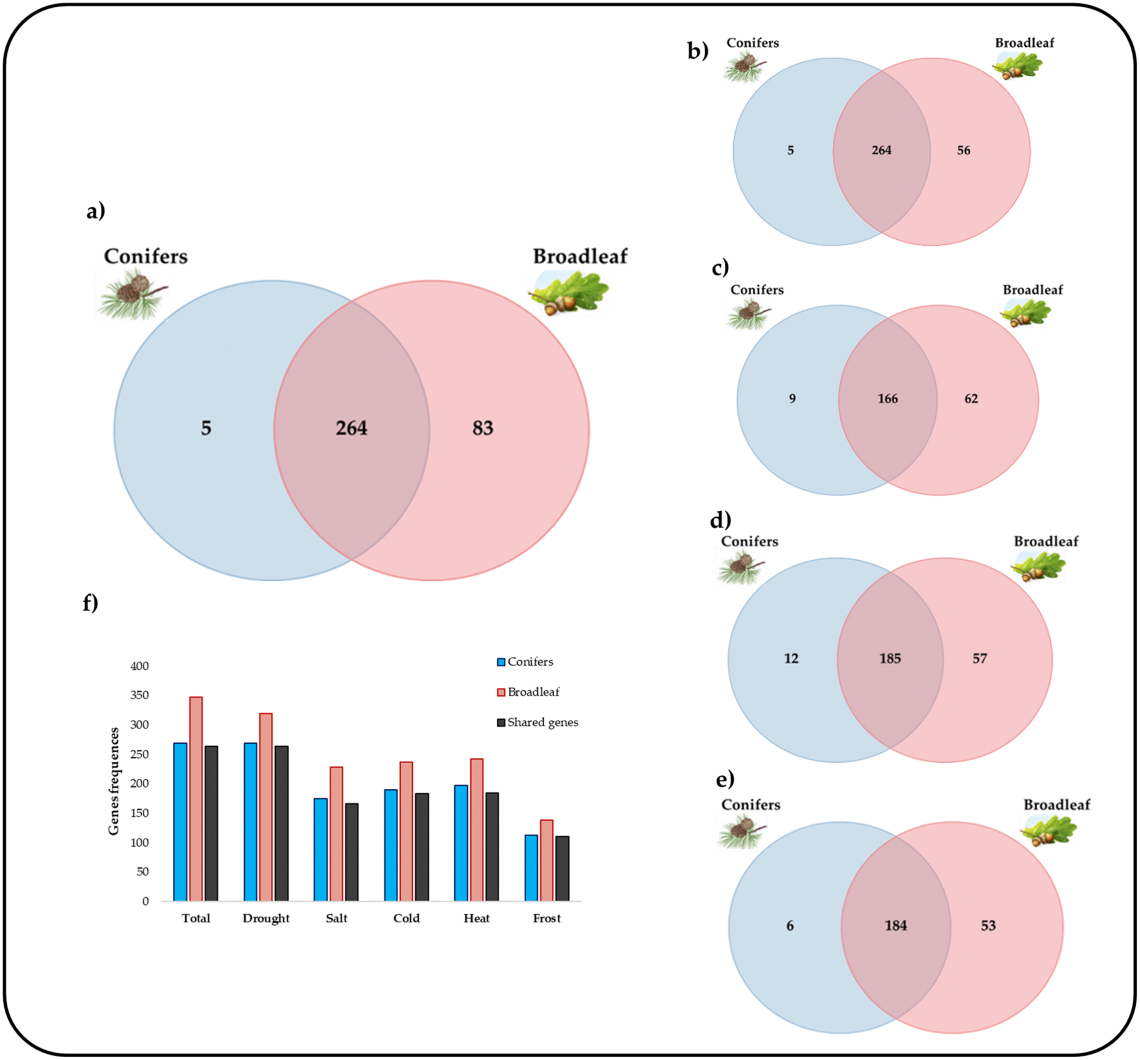
Graphical analysis performed using Venn diagrams. The Venn diagram shows the number of genes belonging to the conifer taxonomic group (blue ensemble) and the angiosperms taxonomic group (red ensemble). The insertion between the two sets represents the number of genes found in common between the two taxonomic groups. The analysis is related to the totality of genes observed (a) and grouped by individual stresses (b-e), drought, salt, heat, cold, and frost, respectively. Figure also shows (f) a histogram representing the absolute frequencies of genes divided by taxonomic groups, shared genes and abiotic stresses studied. Species-specific Venn diagram are reported in S1 File.

For the drought stress response, 264 candidate genes were observed to be present in both angiosperms and coniferous trees reported in the Venn diagrams (Fig 2). We also developed a dataset containing information on only those genes that are possible orthologs between the two observed taxonomic groups (S2 Table).

### 3.3 Multi-alignment and phylogenetic inference

**Fig 3:**
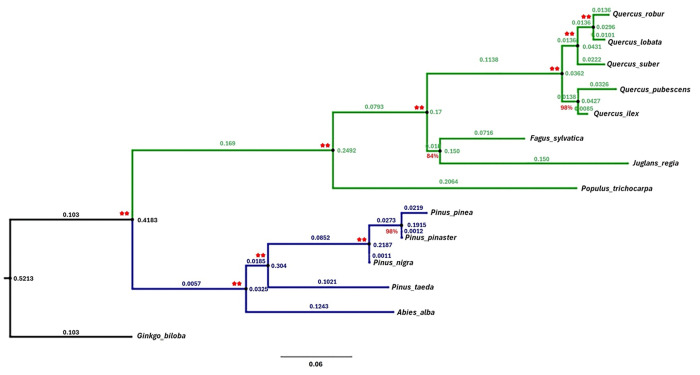
Phylogenetic tree obtained from a partitioned supermatrix-based ML methods regarding the analysis of 264 orthogroups and 13 species distributed between two main seed plant lineages (angiosperms species in green and conifers in blue, with *Ginkgo biloba* L. as outgroup, in black) inferred by various R packages. Double red sign stands for bootstrap supporting of 100%. Otherwise, the percentage of bootstrap supporting is reported. The choice of the substitution model was carried out by using the ModelTest function of the R package Phangorn. Results of the model testing can be found in S4 Table).

A total of 13 species (with the addition of *Ginkgo biloba* L. as outgroup) were used to infer the phylogeny, and 616 protein-coding genes sequences were retrieved and analysed from the taxonomic samples. After the filtering phase, 264 orthogroups, which include all studied species with nucleotide alignment length ranging from 300 to 11,265 bp, were used in the following analyses. The phylogeny reconstructed by partitioned and unpartitioned supermatrix-based methods are topologically identical (S1 and S2 Figs). All tree nodes are supported by a bootstrap value of 100% in the ML tree generated from the partitioned sequences (Fig 3).

Also, in the phylogenetic trees reconstructed from the sequences concatenated by the supermatrix method, all tree nodes are supported by bootstrap values of 100% (S2 Fig).

The phylogenetic analysis carried out reveals that the coniferous and angiosperms groups form two sister clades, indicating a different evolutionary history divided by a speciation event (Fig 3). The structure of the phylogenetic tree reflects those obtained from genomic analysis on similar species [26,34,36,68]. Analyzing the group of angiosperms species, it can be seen that they form a strongly supported monophyletic group (100%), as does that of conifers. The partitioned and unpartitioned methods reported the position of *Ginkgo biloba* L. as an outgroup with high confidence. From the analysis of the monophyletic group of angiosperms species, it can be seen that *Populus trichocarpa* L. is represented by an evolutionary history that diverges from the other species. Here, the genetic distance is 0.2064 with respect to the common ancestor. This could be due to anthropogenic factors, such as poplar domestication [74], or environmental factors, such as the different environmental conditions in which poplar grows, unlike the other species considered. Analysing the monophyletic group of angiosperms species, we can see two sister clades, the one representative of the subfamily *Quercoideae* (genus *Quercus* L.), and the one representative of the subfamily *Juglandaceae* DC. along with the subfamily *Fagoideae* K. Koch. The clade of *Quercoideae* is strongly supported (100%), while the representative clade of *Juglandaceae* and *Fagoideae* is supported by a bootstrap value of 84%. Again, the presence of two distinct clades (that of *Quercoideae* and that of Juglandaceae and *Fagoideae*), could be derived from the different evolutionary history of the groups. In the conifer clade, we can see all tree nodes strongly supported by bootstrap values of 100% (except one that is 98% supported). From the analysis of the monophyletic group of conifers species, it can be seen that *A. alba* is represented by an evolutionary history that diverges from the other species. Here, the genetic distance is 0.1243 with respect to the common ancestor. A monophyletic group can also be observed here, consisting of the representative clade of the genus *Pinus* L, in which *P. taeda* differ from the remaining species (*P. pinea*, *P. pinaster* and *P. nigra*) for 0.4913 mutation per base. This division is supported by other studies [60–62], showing a division between the subgenus *Pinus*, section *Pinus* (*P. pinea*, *P. pinaster* and *P. nigra*), and the subgenus *Pinus*, section *Trifoliae* (P. *taeda*). The *Abietoideae* clade is strongly supported by a bootstrap value of 100%.

### 3.4 Gene concordance analysis

To formally assess the concordance between individual gene trees and the concatenated species tree, we calculated gCF and sCF for each branch of the phylogeny. These metrics quantify the proportion of individual gene trees or alignment sites that support a given branch, while gene discordance factors (gDF1, gDF2, gDFP) capture the degree of conflict. Across the analyzed branches (branch ID 15–25), mean gCF values ranged from 43% to 87%, indicating variable concordance among loci (Table 4; S7 Table). Branches 23 and 22 exhibited the highest concordance, with mean gCF of 87% and 85%, respectively, and over 85% of genes supporting these splits. In contrast, branches 25, 24, and 20 showed the lowest mean gCF values (43–46%), reflecting conflict among individual gene trees. The median gCF was frequently 100% for well-supported branches, highlighting that for many branches at least half of the loci fully agreed with the species tree.

**Table 4:** Gene and site concordance summary for phylogenetic branches. Branch-specific mean gene concordance factors (mean_gCF) and site concordance factors (mean_sCF) are reported, along with the percentage of genes with gCF > 50% (). Gene discordance factors (gDF1, gDF2, gDFP) indicate the proportion of loci supporting alternative topologies.

BranchID	mean_gCF	gCF50	mean_sCF	mean_gDF1	mean_gDF2	mean_gDFP
**15**	75	75	48	2	0	23
**16**	70	70	56	0,53	0	29
**17**	67	67	52	0	0	33
**18**	49	49	43	3	0	48
**19**	52	52	59	3	0,53	44
**20**	46	46	24	31	1	22
**21**	74	74	29	12	2	12
**22**	85	85	40	5	0,54	9
**23**	87	87	81	0,53	1	12
**24**	46	46	37	8	16	30
**25**	43	43	37	15	8	34

The sCF, ranged from 10% (Branch 20) to 81% (Branch 23). High sCF values for branch 23 indicate that the concatenated alignment strongly supports this split, whereas low sCF values for branches 20, 24, and 25 suggest localized site-level conflict.

**Fig 4:**
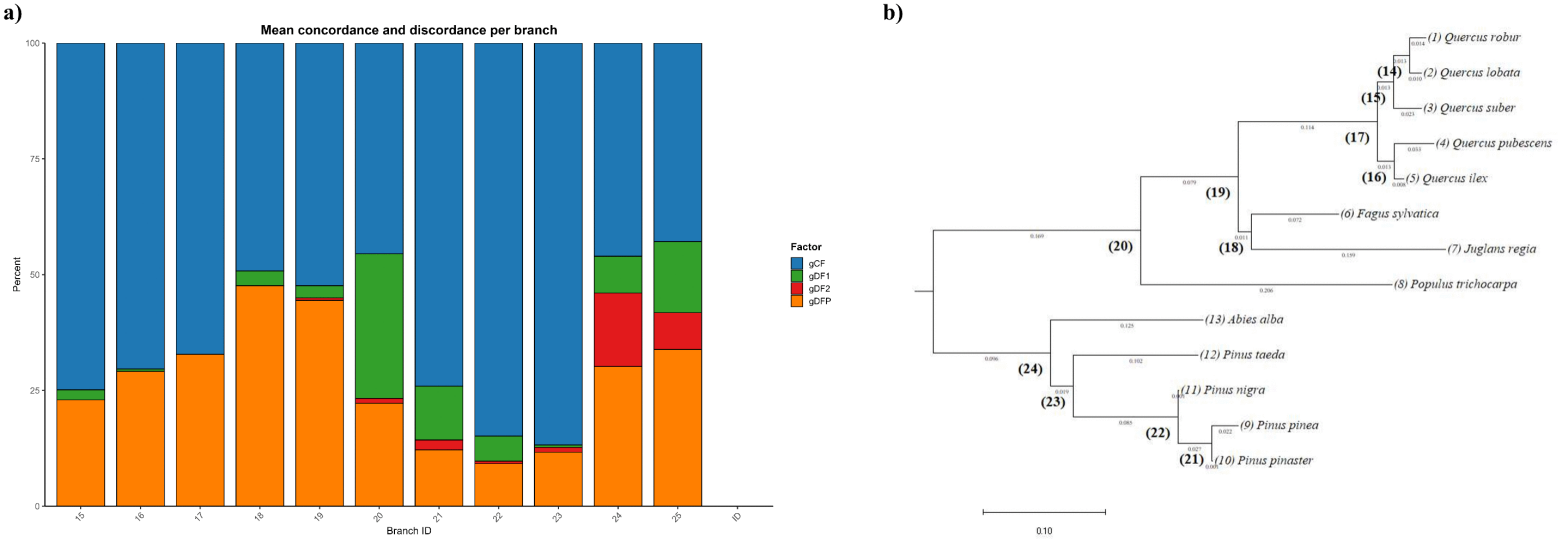
Boxplot showing gene concordance and discordance (a) for the set of orthologous loci used in the phylogenetic inference, and the resulting concatenated phylogenetic tree (b).

Discordance metrics further illustrated these patterns. Branches with lower mean gCF, such as 20, 24, and 25, exhibited elevated gDF1, gDF2, or gDFP values, indicating alternative topologies present among the gene trees. For instance, branch 20 had a mean gDF1 of 31%, consistent with substantial gene-tree discordance in this clade. Conversely, branches with high gCF, such as 22 and 23, showed minimal discordance (gDF1 and gDF2 ≤ 5%). Overall, these results confirm that while the majority of loci are congruent with the concatenated species tree, certain branches exhibit moderate to high gene-tree conflict (Fig 4). This supports the robustness of the concatenated phylogeny, particularly for branches with high gCF and sCF, while highlighting regions where lineage-specific histories or incomplete lineage sorting may contribute to discordance.

### 3.5 Substitution rate in angiosperms and conifers

We aligned and analysed the sequences of the 264 orthogroups found. For each orthogroup analysed, sequences afferent to the 2 taxonomic groups conifers and angiosperms trees were separated and analysed again. We thus obtained and aligned, for the 264 orthogroups considered, 368 sequences for broadleaf and 322 sequences for conifers (S2 Table). The 690 sequences were analysed to infer the number of synonymous (Ks) and nonsynonymous (Ka) substitutions per site (S5 Table and S2 File). The results obtained were used for filtering the analysed sequences to eliminate possible outgroups. We have checked our data for outlier identification as reported by Buschiazzo *et al*. [68]. For conifers, we discarded 57 orthologues in total (45 with Ks > 2.5 and 12 with Ka > 1.5), and for angiosperms species, we discarded 39 genes (26 genes with Ks > 8, 4 genes with Ka > 1.5 and 26 genes with Ka/Ks ratio < 0.09). Thresholds were determined by plotting Ka as a function of Ks and excluding outliers from the main distribution (Additional File3: S4 Table). The final orthologue dataset contained 264 orthogroups (265 conifers genes and 329 angiosperms species genes). The 594 sequences obtained after filtering were analysed to infer the final number of synonymous (Ks) and nonsynonymous (Ka) substitutions per site (Table 5).

**Table 5:** Substitution rates in conifer protein-coding genes compared to angiosperms species protein-coding genes. Mean genetic distances at synonymous (Ks) and non-synonymous (Ka) sites are expressed as a number of substitutions per site.

Pairwise comparison	Gene number	Ks (dS)	Ka (dN)	Ka/Ks (dN/dS)
**Conifers:**	**265**	**0.4610**	**0.1976**	**0.4286**
***Abies alba*** ** Mill.**	*196*			
***Pinus pinea*** ** L.**	*28*			
***Pinus pinaster*** ** Aiton**	*29*			
***Pinus nigra*** ** J.F.Arnold**	*12*			
**Angiosperms:**	**329**	**1.7439**	**0.3847**	**0.2206**
***Fagus sylvatica*** ** L.**	*180*			
***Quercus robur*** ** L.**	*107*			
***Quercus pubescens*** ** Willd.**	*28*			
***Quercus ilex*** ** L.**	*14*			
**Fold change**				
**Angiosperms:Conifers**		**3.78:1**	**1.94:1**	**1:2.09**

**Fig 5:**
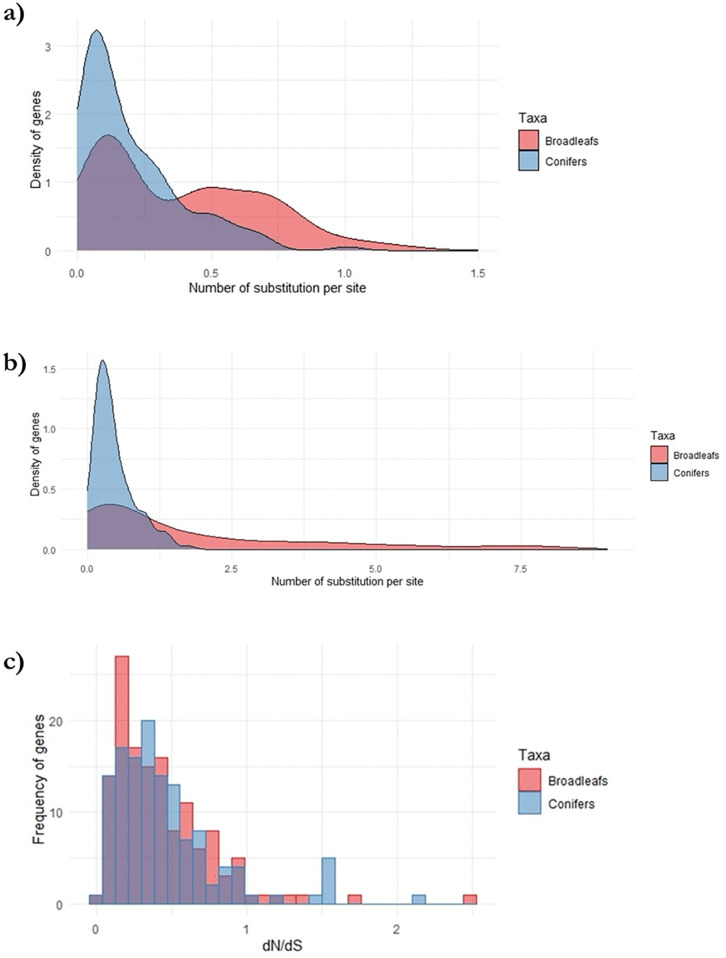
Distribution of evolutionary estimates for conifer and angiosperms protein-coding genes. (a). Smoothed density plots of Ks estimates. (b). Smoothed density plot of Ka estimates. (c). Histogram plots of Ka/Ks estimates.

The mean Ks was 0.4610 for conifers ([CI] = 0.4570, 0.4650), which can be interpreted as the possibility that at least one mutation per 2.2 sites in all lineages from the common ancestor occurred. The average Ks was 1.7439 ([CI] = 1.7401, 1.7482), for angiosperms species. Using the same key, it can be assumed that at least one mutation every 0.57 sites occurred in all lineages, starting with the ancestor anyway. The mean of Ka was lower than that of Ks for both gymnosperms (0.1976; [CI] = 0.1952, 0.1998) and angiosperms species (0.3847; [CI] = 0.3806, 0.3871). The mean Ka value lower than Ks value would be interpretable as an expected mutational constraint on nonsynonymous sites [22]. The Ka/Ks ratio calculated over the entire gene sequence can be used as a rough indication of adaptive evolution [37]. The average Ka/Ks ratio in conifer genes was 0.4286 ([CI] = 0.4223, 0.4302), and 0.2206 ([CI] = 0.2172, 0.2242) for angiosperms species. Only a small percentage (about 5%) of the gene sequences analysed showed a Ka/Ks ratio >1 (S5 and S6 Tables). Comparing the two taxonomic groups studied, it is possible to observe higher average values of Ks and Ka in angiosperms species than in conifers, by 3.78- and 1.94-fold, respectively. Figs 5A and 5B illustrates the difference in Ks and Ka distributions between the two analysed taxonomic groups (conifers and angiosperms species). The average Ka/Ks value was found to be higher in conifers than angiosperms trees by about 2 times (Table 5). Furthermore, Fig 4C shows that the distributions of the Ka/Ks ratios of the two taxonomic groups extend beyond unity, with a greater representation for conifers than angiosperms trees.

## 4 Discussion

The adaptation, diversification, and extinction of higher plant lineages were shaped due to the different sources of environmental cues. The study of abiotic stress response in these lineages has become one of the topics of greatest interest within this discipline. Using sequenced genomes, even of non-model species, some studies aimed to decipher the evolution of entire families and genera [75,76]. Despite the abundance of genomic data, some forest species lack comprehensive phylogeny studies and are absent from environmental association studies, particularly those related to European forest ecosystems. Characterizing these genetic variants enables us to address fundamental questions regarding how evolution influences a species. Specifically, how a species occupies ecological niche can significantly affect the number, size, and genomic distribution of alleles contributing to variation in adaptive traits (i.e., its genetic architecture), as well as the copy number and genomic placement of loci that may influence these traits (i.e., its genomic architecture). As individuals disperse across different regions, they may encounter varying environments or competitors, which can lead to natural selection acting with varying intensity or in different directions within trait space. Depending on the interplay between spatial variation in selection and the migration rate across the landscape, heterogeneous environments can promote local adaptation [5–8], influence the maintenance of standing genetic variation [8], and contribute to speciation [26,36]. In this study, we applied a multi-disciplinary approach to observe the molecular responses of these 13 forest tree species to abiotic stresses and investigate their different evolutionary histories to better understand the causes of divergence. The aim is to understand the influence of the dynamics of local and global environment adaptation on the events that characterized the evolutionary history of these species. We used a specific dataset of 616 genes representative of the response to abiotic stresses (Drought, Heat, Salt, Frost and Cold) and implicated in the adaptation of the observed forest species.

### 4.1 Insights into shared and unique stress response mechanisms

The study of shared adaptive genes between different plant lineages, holds significant importance in understanding the molecular basis of environmental adaptation and evolution [5,6,12,68]. They have evolved similar genetic solutions to these pressures, reflecting either convergent evolution or the retention of ancient, conserved genes [68]. One striking aspect of these shared genes is their conservation across vastly different taxa, implying that some adaptive responses have been maintained since the groups diverged hundreds of millions of years ago [12,68]. The gene sequence dataset obtained in this study can be considered representative for the response to abiotic stresses [77–80]. The systematic review allowed us, moreover, to observe the involvement of almost all the annotated genes in the response to multiple abiotic stresses (S1 Table). According to scientific papers, many types of abiotic stress responses are systemic and potentially confer tolerance to multiple stresses. Some studies carried out on the response to multiple stresses in herbaceous (*Arabidopsis thaliana* (L.) Heynh. and *Oryza sativa* L.) and arboreal (*Populus trichocarpa* L.) species have shown how the plant response represents the result of complex interactions rather than a simple merging of responses to single stresses [81–84]. In addition, Sewelam *et al*. (2014) hypothesized that genes involved in the response to multiple stresses represent possible better candidates for studying patterns of tolerance to environmental stresses [85]. Genes associated with these systemic responses may provide interesting information about the molecular networks underlying stress resistance [81–88]. This concept is further supported by studies comparing the genomes of conifers and broadleaf species, which revealed a substantial set of shared adaptive genes [7,26,36,68]. Despite the evolutionary divergence of conifers and broadleaf species, they inhabit similar environments and face comparable environmental challenges such as temperature extremes and drought [26,36]. Research comparing species like poplar and *Arabidopsis* with conifers (spruce and pine) shows that genes linked to key traits like cold hardiness, drought tolerance, and phenology are conserved across these groups, underscoring the role of systemic mechanisms in stress adaptation [13,15,68]. This conserved genetic architecture across divergent taxa implies that the mechanisms enabling adaptation to multiple stresses are widespread among plant lineages. It is possible to hypothesize that these systemic response mechanisms are conserved among different plant species. It is possible that since the genes responsible for the response to multiple stresses are conserved among different lineages, probable orthologs can be found between the forest species investigated. So, we graphically represented the division of these genes between the target species divided in two taxonomic groups (broadleaf and conifers) [26,36,68]. The result of the Venn diagram (Fig 2) shows us that most of the genes are shared between conifers and angiosperms trees and thus constitute possible orthologs. We have found that the angiosperms species taxonomic group has a higher number of unique genes. The higher number of unique genes detected in angiosperms species compared to conifers can be attributed to several key factors related to their evolutionary history, genome complexity, and adaptation mechanisms [68,89]. Angiosperms generally exhibit faster rates of evolution and diversification compared to gymnosperms [26,36,89]. This is partly due to their relatively shorter generation times and more dynamic reproductive strategies, which lead to more frequent genetic mutations and quicker adaptation to environmental changes [26,89]. This accelerated evolution allows angiosperms to acquire new genes or modify existing ones more rapidly, resulting in a greater number of unique genes. Second, genome duplication events, or polyploidy, are more common in angiosperms [26,36,68,89]. These events result in the duplication of entire sets of genes, providing raw material for evolutionary innovation. Over time, duplicated genes may diverge in function or expression patterns, contributing to the broader genetic diversity observed in angiosperms [68,90]. In contrast, gymnosperms tend to evolve more conservatively, with fewer large-scale genomic changes [36,68,89]. Finally, angiosperms often occupy more diverse and dynamic ecological niches than gymnosperms, leading to the evolution of unique gene sets associated with environmental adaptation [68,89]. Gymnosperms, by contrast, tend to occupy more stable ecological niches, leading to the retention of a more conserved set of adaptive genes. These evolutionary, genomic, and ecological factors explain the observed disparity in the number of unique genes between angiosperms and gymnosperms.

### 4.2 Phylogenetic inference uncovers evolutionary divergence in forest species

In the era of phylogenomic, great progress has been made in elucidating the deep relationships of higher plants, especially angiosperms and gymnosperms [90–93]. A large amount of data from angiosperms has been collected over time, culminating in a highly resolved tree [90]. However, some studies highlighted the presence of some gaps in this scientific topic due to the absence of studies related to adaptive traits (especially among gymnosperms) [19,36,94]. Although using many genes and/or investigating many taxa, current phylogeny work has never used a specific set of nuclear genes associated with abiotic stress response mechanisms and/or to the adaptation to local or regional environment [36,95]. Identifying the rate of evolution and nucleotide substitution patterns underlying DNA evolution must be a fundamental goal of molecular genomics [68]. Using data concerning genes relevant to environmental adaptation, it is possible to observe how random and selective-oriented forces have driven the evolution of forest species. In this work, we used a specific set of genes representative of the response to abiotic stresses in major 13 forest species. The tree results align with the species representation found in the studies by Buschiazzo *et al*. [68] and De la Torre *et al*. [36]. Therefore, albeit with a limited set of nuclear genes, our analyses further support the topologies recovered using comprehensive analyses of plastid genomes (but based on fewer than 100 specimens). However, nuclear-plastid conflicts are prevalent at different taxonomic levels of angiosperms and may reflect the evolutionary complexity of angiosperms, involving distinct evolutionary histories inferred from different genomic sources [96]. Analyzing the topology of the resulting phylogenetic tree reveals a clear distinction between angiosperms species and gymnosperms, which appear as sister clades. An interesting finding is the greater evolutionary rate and diversification of the broadleaf group compared to conifers. Specifically, the monophyletic group of broadleaf species has accumulated 0.169 substitutions per site, in contrast to only 0.0057 substitutions per site for conifers, suggesting a significantly faster evolutionary rate for angiosperms species. Gymnosperms exhibit high levels of genetic diversity within populations but display low rates of nucleotide substitution and speciation. moreover, numerous studies indicate a more pronounced slowdown in evolutionary rates for conifers compared to angiosperms, as evidenced by metrics such as recombination rates, nucleotide diversity, and substitution rates [68]. Some research has reported that angiosperms are highly diversified, whereas gymnosperms exhibit a very low speciation rate [20,26,36,95,97]. The variation in the haploid chromosome number among conifers is minimal (n = 11–13), with only scant evidence of whole-genome duplication and polyploidy [94–98]. In contrast, angiosperms demonstrate a high rate of chromosomal evolution, along with frequent polyploidy events and genome duplications [68].

### 4.3 Differential substitution rates highlight evolutionary patterns in forest species

Studying substitution rates is integral to reconstructing evolutionary histories, identifying adaptive changes, and gaining insights into the genetic basis of molecular responses to abiotic stress in forest species. Synonymous and non-synonymous substitution rates have been found to vary widely within and between taxa [26,36,68]. The interspecies variations are linked to selection pressures on protein structure and function [68]. The appearance of mutations is shaped by the efficacy of DNA repair machinery, life history traits (such as generation time), and metabolic rate. On the other hand, the fixation of mutations over generations is influenced by factors like effective population size, purifying (background) selection, and reproductive strategy. This integrated perspective underscores the complexity of genetic variation, considering both the emergence and persistence of mutations in the evolutionary process. We estimated evolutionary measures for 368 conifer sequences and 322 angiosperms species sequences (representing 268 orthogroups). As shown in Table 5, we found both a smaller Ks and Ka value in conifers than in angiosperms species. We observed that the synonymous substitution rate (Ks) in conifers is significantly lower than in angiosperms (0.4610 *vs*. 1.7439, respectively). Similarly, non-synonymous substitution rates (Ka) are also lower in conifers (0.1976) than in angiosperms (0.3847), though the difference is less pronounced. This suggests that substitutions at synonymous sites are more constrained in conifers, or conversely, non-synonymous sites might be less constrained at comparable mutation rates when compared to angiosperms. Although the exact mechanisms behind these substitution patterns in conifers remain unclear, they likely reflect unique mutational processes and/or selective pressures specific to conifer genes. But what might explain the slower substitution rates observed in conifers? Substitution rates are shaped by both mutation occurrence and fixation within populations [68]. Factors influencing mutation rates include the effectiveness of DNA repair mechanisms, generation times, and metabolic rates. However, little is known about the relative efficiency of DNA repair systems in conifers compared to angiosperms. Additionally, the fixation rate of mutations is governed by genetic drift, purifying selection, and reproductive strategies. The large population sizes and high gene flow typical of conifers are often linked to their low synonymous polymorphism levels [98]. Furthermore, the lower diversification rate in conifers [26,36,51,52] has resulted in fewer speciation-driven bottleneck events compared to angiosperms, maintaining lower genetic diversity across conifer populations. Surprisingly, despite their slow evolutionary rates, conifers exhibit high Ka/Ks ratios. Our analysis of 616 genes indicates that gymnosperms have a higher Ka/Ks ratio than angiosperms, with substitution ratios of 0.2206 for conifers and 0.4286 for angiosperms. These findings suggest a greater likelihood of non-synonymous mutations becoming fixed in conifers than in angiosperms. When examining homologous genes, the ratio of non-synonymous to synonymous substitution provides insights into the strength and mode of selection. A Ka/Ks ratio greater than 1 suggests adaptive selection, while a ratio below 1 indicates purifying selection [68]. Thus, while conifers evolve more slowly overall, their evolutionary dynamics may be shaped by unique selective pressures favouring the retention of non-synonymous changes. In conclusion, we did not observe Ka/Ks values above unity except in a few gene sequences. Some phylogeny works conducted on whole genomes or large sets of gene sequences have reported trends of Ka/Ks ratios above unity for gene sequences involved (GO Term) in stress response [26,36,68]. Following the general assumption that substitutions at synonymous sites in coding regions are selectively neutral, a ratio of Ka/Ks above unity is generally associated with adaptive evolution. However, because of selective constraints imposed on amino acid sites, most genes show a ratio below unity. As a result, even in the presence of positive selection for some sites, a ratio below unity can be obtained. However, it is likely that genes related to abiotic stress response are extremally conserved among lineages since the processes of perception and signal transduction as well as stress response mechanisms are highly conserved throughout the tree of life.

## 5 Conclusions

The adaptive response of forest ecosystems remains a critical area of global scientific inquiry. The ongoing climate crisis presents significant threats to forest species, endangering all life forms within various terrestrial ecosystems. While the adaptive capacity of these organisms is substantial, it is not infinite. The current distribution of these ecosystems reflects a long history of adaptation to environmental fluctuations throughout geological eras. To enhance our understanding of how these mechanisms for mitigating and adapting to environmental stressors have evolved, it is essential to investigate their evolutionary history. In this study, we aimed to analyze the molecular basis of local adaptation by comparing 13 different forest tree species, categorized into two primary clades: conifers and angiosperms species. Our findings reveal a high level of conservation in molecular response mechanisms between these groups. However, our analysis of nucleotide substitution rates indicates that conifer orthologs exhibit slower substitution rates compared to their angiosperms angiosperms counterparts. These results contribute to a deeper understanding regarding how adaptive mutation have shaped the evolutionary history of forest species. Nevertheless, significant advancements in technology are required to enhance genomic resources for major forest species, facilitating a more comprehensive understanding of the evolutionary mechanisms and characteristics that underlie these complex genomes.

## Supporting information

S1 Table Candidate genes dataset. List of candidate genes for the target species.
(XLSX)

S2 Table Shared candidate genes. List of gene ID after Venn diagram shared gene detection.
(XLSX)

S3 Table Phylogenetic inference analysis. Shimodaira-Hasegawa test for phylogenetic inference significance.
(XLSX)

S4 Table Substitution model choice. Akaike Information Criteria and Baeyesian Information Criteria.
(XLSX)

S5 Table Synonymous/Non-synonymous substitution rate. Values for the coefficients Ka, Ks, l0, l2, l4, and Ka/Ks ratio for conifers and broadleaves.
(XLSX)

S6 Table Threshold calculation by plotting Ka as a function of K. (XLSX)

S7 Table Gene concordance analysis. (XLSX)

S1 Fig Phylogenetic tree. Phylogenetic tree (partitioned supermatrix-based analysis).
(PNG)

S2 Fig Phylogenetic tree. Phylogenetic tree (un-partitioned supermatrix-based analysis).
(PNG)

S1 File Species-specific Venn diagram. (DOCX)

S2 File Substitution rate analysis. Values of Substitution rate and graphical representation of substitution per site number in the analysed Phylogenetic groups.
(DOCX)

## References

[1] Allen CD, Macalady AK, Chenchouni H, Bachelet D, McDowell N, Vennetier M, et al. A global overview of drought and heat-induced tree mortality reveals emerging climate change risks for forests. Forest Ecol Manag. 2010;259(4):660–84. doi: 10.1016/j.foreco.2009.09.001

[2] Lefèvre F, Boivin T, Bontemps A, Courbet F, Davi H, Durand-Gillmann M, et al. Considering evolutionary processes in adaptive forestry. Ann Forest Sci. 2013;71(7):723–39. doi: 10.1007/s13595-013-0272-1

[3] Chen Z-H, Soltis DE. Evolution of environmental stress responses in plants. Plant Cell Environ. 2020;43(12):2827–31. doi: 10.1111/pce.13922 33103798

[4] Yang Y, Yang Z, Ferguson DK. The Systematics and Evolution of Gymnosperms with an Emphasis on a Few Problematic Taxa. Plants (Basel). 2024;13(16):2196. doi: 10.3390/plants13162196 39204632 PMC11360501

[5] Neale DB, Martínez-García PJ, De La Torre AR, Montanari S, Wei X-X. Novel Insights into Tree Biology and Genome Evolution as Revealed Through Genomics. Annu Rev Plant Biol. 2017;68:457–83. doi: 10.1146/annurev-arplant-042916-041049 28226237

[6] Lind BM, Menon M, Bolte CE, Faske TM, Eckert AJ. The genomics of local adaptation in trees: are we out of the woods yet? Tree Gen Genom. 2018;14(2). doi: 10.1007/s11295-017-1224-y

[7] Bromham L, Hua X, Cardillo M. Macroevolutionary and macroecological approaches to understanding the evolution of stress tolerance in plants. Plant Cell Environ. 2020;43(12):2832–46. doi: 10.1111/pce.13857 32705700

[8] Yeaman S. Evolution of polygenic traits under global vs local adaptation. Genetics. 2022;220(1):iyab134. doi: 10.1093/genetics/iyab134 35134196 PMC8733419

[9] Des Marais DL, Juenger TE. Pleiotropy, plasticity, and the evolution of plant abiotic stress tolerance. Ann N Y Acad Sci. 2010;1206:56–79. doi: 10.1111/j.1749-6632.2010.05703.x 20860683

[10] De La Torre AR, Birol I, Bousquet J, Ingvarsson PK, Jansson S, Jones SJM, et al. Insights into conifer giga-genomes. Plant Physiol. 2014;166(4):1724–32. doi: 10.1104/pp.114.248708 25349325 PMC4256843

[11] De Storme N, Geelen D. The impact of environmental stress on male reproductive development in plants: biological processes and molecular mechanisms. Plant Cell Environ. 2014;37(1):1–18. doi: 10.1111/pce.12142 23731015 PMC4280902

[12] Bromham L, Hua X, Cardillo M. Detecting Macroevolutionary Self-Destruction from Phylogenies. Syst Biol. 2016;65(1):109–27. doi: 10.1093/sysbio/syv062 26454872

[13] Zhao C, Wang Y, Chan KX, Marchant DB, Franks PJ, Randall D, et al. Evolution of chloroplast retrograde signaling facilitates green plant adaptation to land. Proc Natl Acad Sci U S A. 2019;116(11):5015–20. doi: 10.1073/pnas.1812092116 30804180 PMC6421419

[14] Billah M, Aktar S, Brestic M, Zivcak M, Khaldun ABM, Uddin MS, et al. Progressive Genomic Approaches to Explore Drought- and Salt-Induced Oxidative Stress Responses in Plants under Changing Climate. Plants (Basel). 2021;10(9):1910. doi: 10.3390/plants10091910 34579441 PMC8471759

[15] Angon PB, Tahjib-Ul-Arif M, Samin SI, Habiba U, Hossain MA, Brestic M. How Do Plants Respond to Combined Drought and Salinity Stress?-A Systematic Review. Plants (Basel). 2022;11(21):2884. doi: 10.3390/plants11212884 36365335 PMC9655390

[16] dos Santos TB, Ribas AF, de Souza SGH, Budzinski IGF, Domingues DS. Physiological Responses to Drought, Salinity, and Heat Stress in Plants: A Review. Stresses. 2022;2(1):113–35. doi: 10.3390/stresses2010009

[17] Li S, Lu S, Wang J, Chen Z, Zhang Y, Duan J, et al. Responses of Physiological, Morphological and Anatomical Traits to Abiotic Stress in Woody Plants. Forests. 2023;14(9):1784. doi: 10.3390/f14091784

[18] Soltis PS, Soltis DE. Plant genomes: Markers of evolutionary history and drivers of evolutionary change. Plants People Planet. 2020;3(1):74–82. doi: 10.1002/ppp3.10159

[19] Plomion C, Bastien C, Bogeat-Triboulot MB, Bouffier L, Déjardin A, Duplessis S, et al. Forest tree genomics: 10 achievements from the past 10 years and future prospects. Ann Forest Sci. 2016;73:77–103. doi: 10.1007/s13595-015-0488-3

[20] Savolainen O, Pyhäjärvi T, Knürr T. Gene Flow and Local Adaptation in Trees. Annu Rev Ecol Evol Syst. 2007;38(1):595–619. doi: 10.1146/annurev.ecolsys.38.091206.095646

[21] Alberto FJ, Aitken SN, Alía R, González‐Martínez SC, Hänninen H, Kremer A, et al. Potential for evolutionary responses to climate change – evidence from tree populations. Glob Change Biol. 2013;19(6):1645–61. doi: 10.1111/gcb.12181 PMC3664019 23505261

[22] Sork VL, Aitken SN, Dyer RJ, Eckert AJ, Legendre P, Neale DB. Putting the landscape into the genomics of trees: approaches for understanding local adaptation and population responses to changing climate. Tree Gen Genom. 2013;9(4):901–11. doi: 10.1007/s11295-013-0596-x

[23] Boshier D, Broadhurst L, Cornelius J, Gallo L, Koskela J, Loo J, et al. Is local best? Examining the evidence for local adaptation in trees and its scale. Environ Evid. 2015;4(1). doi: 10.1186/s13750-015-0046-3

[24] Prunier J, Laroche J, Beaulieu J, Bousquet J. Scanning the genome for gene SNPs related to climate adaptation and estimating selection at the molecular level in boreal black spruce. Mol Ecol. 2011;20(8):1702–16. doi: 10.1111/j.1365-294X.2011.05045.x 21375634

[25] Holliday JA, Aitken SN, Cooke JEK, Fady B, González-Martínez SC, Heuertz M, et al. Advances in ecological genomics in forest trees and applications to genetic resources conservation and breeding. Mol Ecol. 2017;26(3):706–17. doi: 10.1111/mec.13963 27997049

[26] De La Torre AR, Li Z, Van de Peer Y, Ingvarsson PK. Contrasting Rates of Molecular Evolution and Patterns of Selection among Gymnosperms and Flowering Plants. Mol Biol Evol. 2017;34(6):1363–77. doi: 10.1093/molbev/msx069 28333233 PMC5435085

[27] Isabel N, Holliday JA, Aitken SN. Forest genomics: Advancing climate adaptation, forest health, productivity, and conservation. Evol Appl. 2019;13(1):3–10. doi: 10.1111/eva.12902 31892941 PMC6935596

[28] Leitch AR, Leitch IJ. Ecological and genetic factors linked to contrasting genome dynamics in seed plants. New Phytol. 2012;194(3):629–46. doi: 10.1111/j.1469-8137.2012.04105.x 22432525

[29] Morse AM, Peterson DG, Islam-Faridi MN, Smith KE, Magbanua Z, Garcia SA, et al. Evolution of genome size and complexity in Pinus. PLoS One. 2009;4(2):e4332. doi: 10.1371/journal.pone.0004332 19194510 PMC2633040

[30] Nystedt B, Street NR, Wetterbom A, Zuccolo A, Lin Y-C, Scofield DG, et al. The Norway spruce genome sequence and conifer genome evolution. Nature. 2013;497(7451):579–84. doi: 10.1038/nature12211 23698360

[31] Prunier J, Verta J-P, MacKay JJ. Conifer genomics and adaptation: at the crossroads of genetic diversity and genome function. New Phytol. 2016;209(1):44–62. doi: 10.1111/nph.13565 26206592

[32] Neale DB, Kremer A. Forest tree genomics: growing resources and applications. Nat Rev Genet. 2011;12(2):111–22. doi: 10.1038/nrg2931 21245829

[33] Heslop-Harrison JSP, Schwarzacher T, Liu Q. Polyploidy: its consequences and enabling role in plant diversification and evolution. Ann Bot. 2023;131(1):1–10. doi: 10.1093/aob/mcac132 36282971 PMC9904344

[34] Folk RA, Siniscalchi CM, Soltis DE. Angiosperms at the edge: Extremity, diversity, and phylogeny. Plant Cell Environ. 2020;43(12):2871–93. doi: 10.1111/pce.13887 32926444

[35] Zhou S-S, Xing Z, Liu H, Hu X-G, Gao Q, Xu J, et al. In-depth transcriptome characterization uncovers distinct gene family expansions for Cupressus gigantea important to this long-lived species’ adaptability to environmental cues. BMC Genomics. 2019;20(1):213. doi: 10.1186/s12864-019-5584-6 30866823 PMC6417167

[36] De La Torre AR, Piot A, Liu B, Wilhite B, Weiss M, Porth I. Functional and morphological evolution in gymnosperms: A portrait of implicated gene families. Evol Appl. 2019;13(1):210–27. doi: 10.1111/eva.12839 31892953 PMC6935586

[37] Roodt D, Lohaus R, Sterck L, Swanepoel RL, Van de Peer Y, Mizrachi E. Evidence for an ancient whole genome duplication in the cycad lineage. PLoS One. 2017;12(9):e0184454. doi: 10.1371/journal.pone.0184454 28886111 PMC5590961

[38] Young AD, Gillung JP. Phylogenomics — principles, opportunities and pitfalls of big‐data phylogenetics. Syst Entomol. 2019;45(2):225–47. doi: 10.1111/syen.12406

[39] Lotterhos KE, Whitlock MC. Evaluation of demographic history and neutral parameterization on the performance of FST outlier tests. Mol Ecol. 2014;23(9):2178–92. doi: 10.1111/mec.12725 24655127 PMC4228763

[40] Savolainen O, Lascoux M, Merilä J. Ecological genomics of local adaptation. Nat Rev Genet. 2013;14(11):807–20. doi: 10.1038/nrg3522 24136507

[41] Platt A, Vilhjálmsson BJ, Nordborg M. Conditions Under Which Genome-Wide Association Studies Will be Positively Misleading. Genetics. 2010;186(3):1045–52. doi: 10.1534/genetics.110.121665 20813880 PMC2975277

[42] Pritchard JK, Pickrell JK, Coop G. The genetics of human adaptation: hard sweeps, soft sweeps, and polygenic adaptation. Curr Biol. 2010;20(4):R208-15. doi: 10.1016/j.cub.2009.11.055 20178769 PMC2994553

[43] Pritchard JK, Di Rienzo A. Adaptation - not by sweeps alone. Nat Rev Genet. 2010;11(10):665–7. doi: 10.1038/nrg2880 20838407 PMC4652788

[44] Hermisson J, Pennings PS. Soft sweeps and beyond: understanding the patterns and probabilities of selection footprints under rapid adaptation. Methods Ecol Evol. 2017;8(6):700–16. doi: 10.1111/2041-210x.12808

[45] Galtier N. Adaptive Protein Evolution in Animals and the Effective Population Size Hypothesis. PLoS Genet. 2016;12(1):e1005774. doi: 10.1371/journal.pgen.1005774 26752180 PMC4709115

[46] Booker T, Yeaman S, Whitlock MC. Global adaptation confounds the search for local adaptation. Evol Lett. 2021;5:4–15. doi: 10.1002/evl3.206 33552532 PMC7857299

[47] Booker TR, Jackson BC, Keightley PD. Detecting positive selection in the genome. BMC Biol. 2017;15(1):98. doi: 10.1186/s12915-017-0434-y 29084517 PMC5662103

[48] Rockman MV. The QTN program and the alleles that matter for evolution: all that’s gold does not glitter. Evolution. 2012;66(1):1–17. doi: 10.1111/j.1558-5646.2011.01486.x 22220860 PMC3386609

[49] Yeaman S. Local Adaptation by Alleles of Small Effect. Am Nat. 2015;186:S74–89. doi: 10.1086/682405 26656219

[50] Stephan W. Selective Sweeps. Genetics. 2019;211(1):5–13. doi: 10.1534/genetics.118.301319 30626638 PMC6325696

[51] Baker WJ, Bailey P, Barber V, Barker A, Bellot S, Bishop D, et al. A Comprehensive Phylogenomic Platform for Exploring the Angiosperm Tree of Life. Syst Biol. 2022;71(2):301–19. doi: 10.1093/sysbio/syab035 33983440 PMC8830076

[52] Paterson AH, Freeling M, Tang H, Wang X. Insights from the Comparison of Plant Genome Sequences. Annu Rev Plant Biol. 2010;61(1):349–72. doi: 10.1146/annurev-arplant-042809-112235 20441528

[53] Johnson M, Zaretskaya I, Raytselis Y, Merezhuk Y, McGinnis S, Madden TL. NCBI BLAST: a better web interface. Nucleic Acids Res. 2008;36(Web Server issue):W5–9. doi: 10.1093/nar/gkn201 18440982 PMC2447716

[54] Katoh K, Standley DM. MAFFT multiple sequence alignment software version 7: improvements in performance and usability. Mol Biol Evol. 2013;30(4):772–80. doi: 10.1093/molbev/mst010 23329690 PMC3603318

[55] Kumar S, Stecher G, Li M, Knyaz C, Tamura K. MEGA X: Molecular Evolutionary Genetics Analysis across Computing Platforms. Mol Biol Evol. 2018;35(6):1547–9. doi: 10.1093/molbev/msy096 29722887 PMC5967553

[56] Garosi C, Ferrante R, Vettori C, Paffetti D. Meta-Analysis as a Tool to Identify Candidate Genes Involved in the Fagus sylvatica L. Abiotic Stress Response. Forests. 2022;13(2):159. doi: 10.3390/f13020159

[57] Horiike T, Minai R, Miyata D, Nakamura Y, Tateno Y. Ortholog-Finder: A Tool for Constructing an Ortholog Data Set. Genome Biol Evol. 2016;8(2):446–57. doi: 10.1093/gbe/evw005 26782935 PMC4779612

[58] Ran J-H, Shen T-T, Wu H, Gong X, Wang X-Q. Phylogeny and evolutionary history of Pinaceae updated by transcriptomic analysis. Mol Phylogenet Evol. 2018;129:106–16. doi: 10.1016/j.ympev.2018.08.011 30153503

[59] Chen H, Boutros PC. VennDiagram: a package for the generation of highly-customizable Venn and Euler diagrams in R. BMC Bioinformatics. 2011;12:35. doi: 10.1186/1471-2105-12-35 21269502 PMC3041657

[60] Mosca E, Cruz F, Gómez-Garrido J, Bianco L, Rellstab C, Brodbeck S, et al. A Reference Genome Sequence for the European Silver Fir (Abies alba Mill.): A Community-Generated Genomic Resource. G3 (Bethesda). 2019;9(7):2039–49. doi: 10.1534/g3.119.400083 31217262 PMC6643874

[61] Minh BQ, Schmidt HA, Chernomor O, Schrempf D, Woodhams MD, von Haeseler A, et al. IQ-TREE 2: New Models and Efficient Methods for Phylogenetic Inference in the Genomic Era. Mol Biol Evol. 2020;37(5):1530–4. doi: 10.1093/molbev/msaa015 32011700 PMC7182206

[62] Schliep KP. phangorn: phylogenetic analysis in R. Bioinformatics. 2011;27(4):592–3. doi: 10.1093/bioinformatics/btq706 21169378 PMC3035803

[63] Paradis E, Claude J, Strimmer K. APE: Analyses of Phylogenetics and Evolution in R language. Bioinformatics. 2004;20(2):289–90. doi: 10.1093/bioinformatics/btg412 14734327

[64] Jombart T, Archer F, Schliep K, Kamvar Z, Harris R, Paradis E, et al. apex: phylogenetics with multiple genes. Mol Ecol Resour. 2017;17(1):19–26. doi: 10.1111/1755-0998.12567 27417145 PMC5215480

[65] Guan R, Zhao Y, Zhang H, Fan G, Liu X, Zhou W, et al. Draft genome of the living fossil Ginkgo biloba. GigaScience. 2016;5(1):49. doi: 10.1186/s13742-016-0154-1 27871309 PMC5118899

[66] Goldman N, Yang Z. A codon-based model of nucleotide substitution for protein-coding DNA sequences. Mol Biol Evol. 1994;11(5):725–36. doi: 10.1093/oxfordjournals.molbev.a040153 7968486

[67] Bielawski JP, Yang Z. Maximum Likelihood Methods for Detecting Adaptive Protein Evolution. In: Statistical Methods in Molecular Evolution. Statistics for Biology and Health. New York (NY): Springer; 2005. p. 103–24.

[68] Buschiazzo E, Ritland C, Bohlmann J, Ritland K. Slow but not low: genomic comparisons reveal slower evolutionary rate and higher dN/dS in conifers compared to angiosperms. BMC Evol Biol. 2012;12:8. doi: 10.1186/1471-2148-12-8 22264329 PMC3328258

[69] Kirby KN, Gerlanc D. BootES: an R package for bootstrap confidence intervals on effect sizes. Behav Res Methods. 2013;45(4):905–27. doi: 10.3758/s13428-013-0330-5 23519455

[70] Leebens-Mack JH, Barker MS, Carpenter EJ, Deyholos MK, Gitzendanner MA, Graham SW, et al. One thousand plant transcriptomes and the phylogenomics of green plants. Nature. 2019; 574:679–85. doi: 10.1038/s41586-019-1693-2 31645766 PMC6872490

[71] Altenhoff AM, Dessimoz C. Phylogenetic and functional assessment of orthologs inference projects and methods. PLoS Comput Biol. 2009;5(1):e1000262. doi: 10.1371/journal.pcbi.1000262 19148271 PMC2612752

[72] Gabaldón T. Large-scale assignment of orthology: back to phylogenetics? Genome Biol. 2008;9(10):235. doi: 10.1186/gb-2008-9-10-235 18983710 PMC2760865

[73] Chen M-Y, Liang D, Zhang P. Selecting Question-Specific Genes to Reduce Incongruence in Phylogenomics: A Case Study of Jawed Vertebrate Backbone Phylogeny. Syst Biol. 2015;64(6):1104–20. doi: 10.1093/sysbio/syv059 26276158

[74] Bradshaw HD Jr. Case history in genetics of long-lived plants: molecular approaches to domestication of a fast-growing forest tree: *Populus*. In: Paterson AH, editor. Molecular Dissection of Complex Traits. CRC Press; 1998. p. 219–28.

[75] Ahuja MR, Neale DB. Evolution of Genome Size in Conifers. Silvae Genetica. 2005;54(1–6):126–37. doi: 10.1515/sg-2005-0020

[76] Pelgas B, Beauseigle S, Acheré V, Jeandroz S, Bousquet J, Isabel N. Comparative genome mapping among Picea glauca, *P. mariana* x *P. rubens* and *P. abies*, and correspondence with other *Pinaceae*. Theor Appl Genet. 2006;113(8):1371–93. doi: 10.1007/s00122-006-0354-7 17061103

[77] Müller M, Gailing O. Abiotic genetic adaptation in the Fagaceae. Plant Biol (Stuttg). 2019;21(5):783–95. doi: 10.1111/plb.13008 31081234

[78] Lalagüe H, Csilléry K, Oddou-Muratorio S, Safrana J, de Quattro C, Fady B, et al. Nucleotide diversity and linkage disequilibrium at 58 stress response and phenology candidate genes in a European beech (Fagus sylvatica L.) population from southeastern France. Tree Genet Gen. 2013;10(1):15–26. doi: 10.1007/s11295-013-0658-0

[79] Konopka-Postupolska D, Dobrowolska G. ABA perception is modulated by membrane receptor-like kinases. J Exp Bot. 2020;71(4):1210–4. doi: 10.1093/jxb/erz531 32076729 PMC7031077

[80] Perdiguero P, Barbero MDC, Cervera MT, Collada C, Soto A. Molecular response to water stress in two contrasting Mediterranean pines (*Pinus pinaster* and *Pinus pinea*). Plant Physiol Biochem. 2013;67:199–208. doi: 10.1016/j.plaphy.2013.03.008 23583937

[81] Mittler R. Oxidative stress, antioxidants and stress tolerance. Trends Plant Sci. 2002;7(9):405–10. doi: 10.1016/s1360-1385(02)02312-9 12234732

[82] Noctor G, Mhamdi A, Foyer CH. The roles of reactive oxygen metabolism in drought: not so cut and dried. Plant Physiol. 2014;164(4):1636–48. doi: 10.1104/pp.113.233478 24715539 PMC3982730

[83] Barakat A, Choi A, Yassin NBM, Park JS, Sun Z, Carlson JE. Comparative genomics and evolutionary analyses of the O-methyltransferase gene family in *Populus*. Gene. 2011;479(1–2):37–46. doi: 10.1016/j.gene.2011.02.008 21338660

[84] Comparative Genomics of The Shikimate Pathway in Arabidopsis, *Populus Trichocarpa* and *Oryza Sativa*: Shikimate Pathway Gene Family Structure and Identification of Candidates for Missing Links in Phenylalanine Biosynthesis. Recent Adv Phytochem. 2006;40:85–113. doi: 10.1016/s0079-9920(06)80038-9

[85] Sewelam N, Oshima Y, Mitsuda N, Ohme-Takagi M. A step towards understanding plant responses to multiple environmental stresses: a genome-wide study. Plant Cell Environ. 2014;37(9):2024–35. doi: 10.1111/pce.12274 24417440

[86] White CR, Frangos JA. The shear stress of it all: the cell membrane and mechanochemical transduction. Philos Trans R Soc Lond B Biol Sci. 2007;362(1484):1459–67. doi: 10.1098/rstb.2007.2128 17569643 PMC2440408

[87] Hasegawa PM, Bressan RA, Zhu J-K, Bohnert HJ. Plant cellular and molecular responses to high salinity. Annu Rev Plant Physiol Plant Mol Biol. 2000;51:463–99. doi: 10.1146/annurev.arplant.51.1.463 15012199

[88] Ghassemi-Golezani K, Ghassemi S, Bandehhagh A. Effects of water supply on field performance of chickpea (*Cicer arietinum* L.) cultivars. Int J Agron Plant Product. 2013;4:94–7.

[89] Li H-T, Yi T-S, Gao L-M, Ma P-F, Zhang T, Yang J-B, et al. Origin of angiosperms and the puzzle of the Jurassic gap. Nat Plants. 2019;5(5):461–70. doi: 10.1038/s41477-019-0421-0 31061536

[90] Li H-T, Luo Y, Gan L, Ma P-F, Gao L-M, Yang J-B, et al. Plastid phylogenomic insights into relationships of all flowering plant families. BMC Biol. 2021;19(1):232. doi: 10.1186/s12915-021-01166-2 34711223 PMC8555322

[91] Xue J, Dong S, Wang M, Song T, Zhou G, Li Z, et al. Mitochondrial genes from 18 angiosperms fill sampling gaps for phylogenomic inferences of the early diversification of flowering plants. J Syt Evol. 2021;60(4):773–88. doi: 10.1111/jse.12708

[92] Baker WJ, Bailey P, Barber V, Barker A, Bellot S, Bishop D, et al. A Comprehensive Phylogenomic Platform for Exploring the Angiosperm Tree of Life. Syst Biol. 2021;71(2):301–19. doi: 10.1093/sysbio/syab035 PMC8830076 33983440

[93] Guo T, Mu Q, Wang J, Vanous AE, Onogi A, Iwata H, et al. Dynamic effects of interacting genes underlying rice flowering-time phenotypic plasticity and global adaptation. Genome Res. 2020;30(5):673–83. doi: 10.1101/gr.255703.119 32299830 PMC7263186

[94] Wang C, McCormack ML, Guo D, Li J. Global meta‐analysis reveals different patterns of root tip adjustments by angiosperm and gymnosperm trees in response to environmental gradients. J Biogeograp. 2018;46(1):123–33. doi: 10.1111/jbi.13472

[95] Guo C, Luo Y, Gao L-M, Yi T-S, Li H-T, Yang J-B, et al. Phylogenomics and the flowering plant tree of life. J Integr Plant Biol. 2023;65(2):299–323. doi: 10.1111/jipb.13415 36416284

[96] Soltis DE, Smith SA, Cellinese N, Wurdack KJ, Tank DC, Brockington SF, et al. Angiosperm phylogeny: 17 genes, 640 taxa. Am J Bot. 2011;98(4):704–30. doi: 10.3732/ajb.1000404 21613169

[97] Jaramillo-Correa JP, Verdú M, González-Martínez SC. The contribution of recombination to heterozygosity differs among plant evolutionary lineages and life-forms. BMC Evol Biol. 2010;10:22. doi: 10.1186/1471-2148-10-22 20100325 PMC2826329

[98] Savolainen O, Pyhäjärvi T. Genomic diversity in forest trees. Curr Opin Plant Biol. 2007;10(2):162–7. doi: 10.1016/j.pbi.2007.01.011 17292660

