# Supplementary figures and images for: Investigating adaptation to environmental variability in forest trees through molecular phylogenetic analysis
Source: PLoS One. 2025 Dec 23;20(12):e0338893. doi: 10.1371/journal.pone.0338893 (PMC12725590; doi:10.1371/journal.pone.0338893)

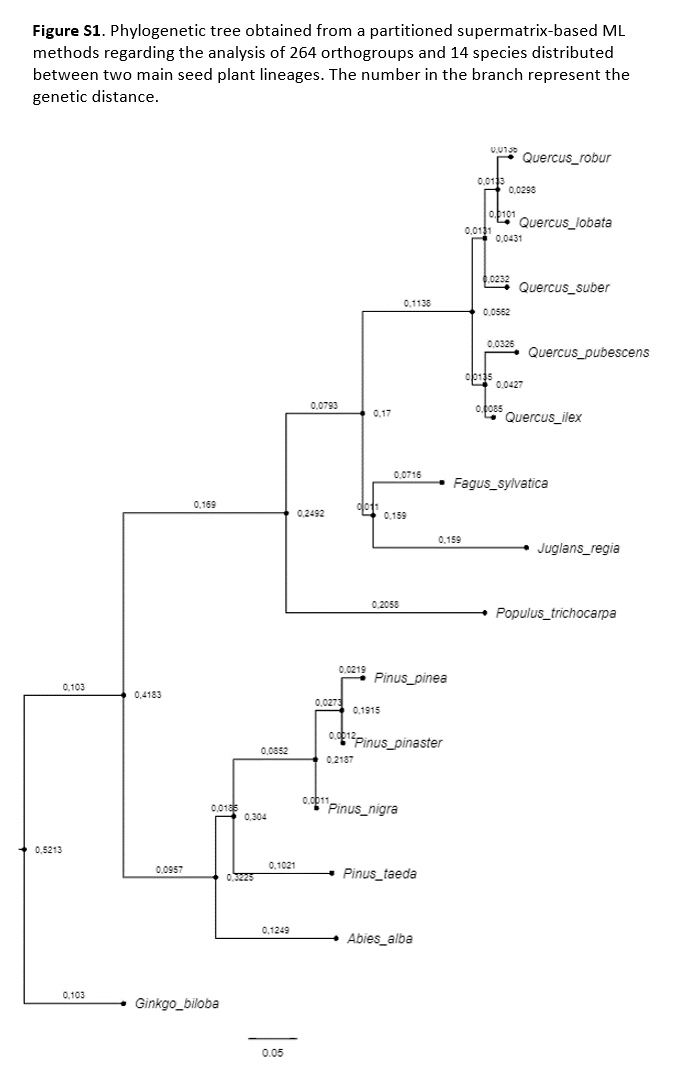

Supplement: S1 Fig — Phylogenetic tree (partitioned supermatrix-based analysis). (PNG) [file pone.0338893.s008.png]

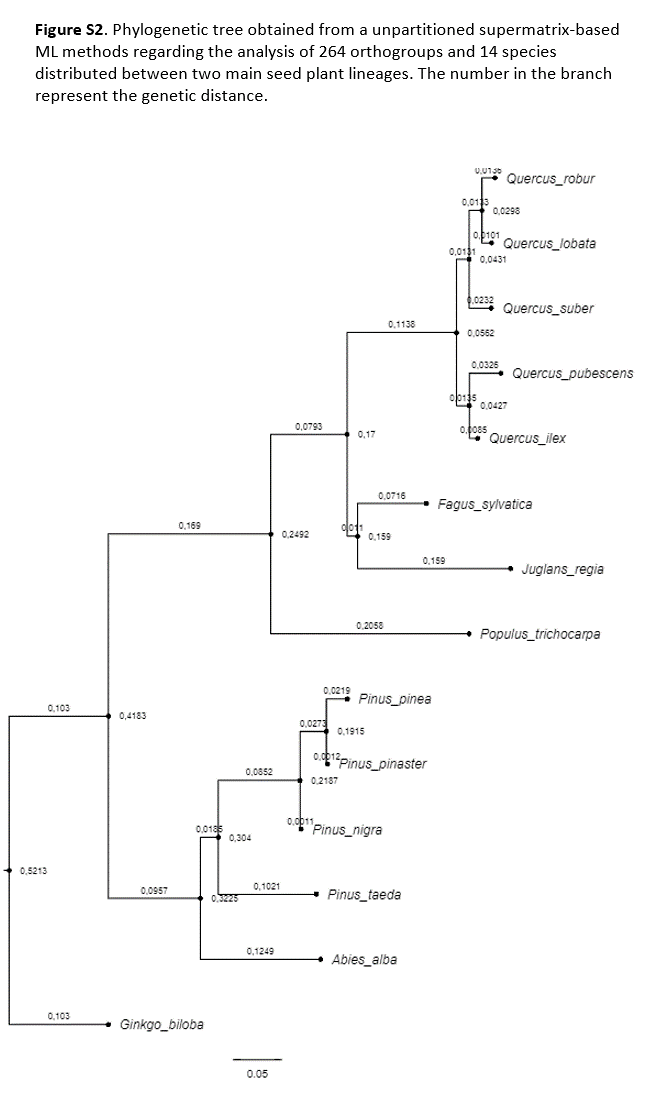

Supplement: S2 Fig — Phylogenetic tree (un-partitioned supermatrix-based analysis). (PNG) [file pone.0338893.s009.png]
