# Supplementary material for: Investigating adaptation to environmental variability in forest trees through molecular phylogenetic analysis
Source: PLoS One. 2025 Dec 23;20(12):e0338893. doi: 10.1371/journal.pone.0338893 (PMC12725590; doi:10.1371/journal.pone.0338893)
Supplement: S1 File — (DOCX) [file pone.0338893.s010.docx]

Investigating adaptation to environmental variability in forest trees through molecular phylogenetic analysis

Cesare Garosi^1#a^, Cristina Vettori^1,2*^, Roberta Ferrante^1,3^, Donatella Paffetti1^1,3^

^1^ Department of Agriculture, Food, Environment and Forestry (DAGRI), University of Florence, Piazzale delle Cascine 18, 50144 Florence, Italy

^2^ National Research Council of Italy (CNR), Institute of Bioscience and Bioresources (IBBR), Division of Florence, Via Madonna del Piano 10, 50019 Sesto Fiorentino, Italy

^3^ NBFC, National Biodiversity Future Center, Palermo 90133, Italy

^#a^Current address: Institute of Research on Terrestrial Ecosystems (IRET), National Research Council of Italy (CNR), Via Madonna del Piano 10, 50019 Sesto Fiorentino, Italy

*** Corresponding author:**

E-mail: [cristina.vettori@cnr.it](mailto:cristina.vettori@cnr.it) (CV)

S1 Appendix: **Venn diagram related to each species separately**

**S1
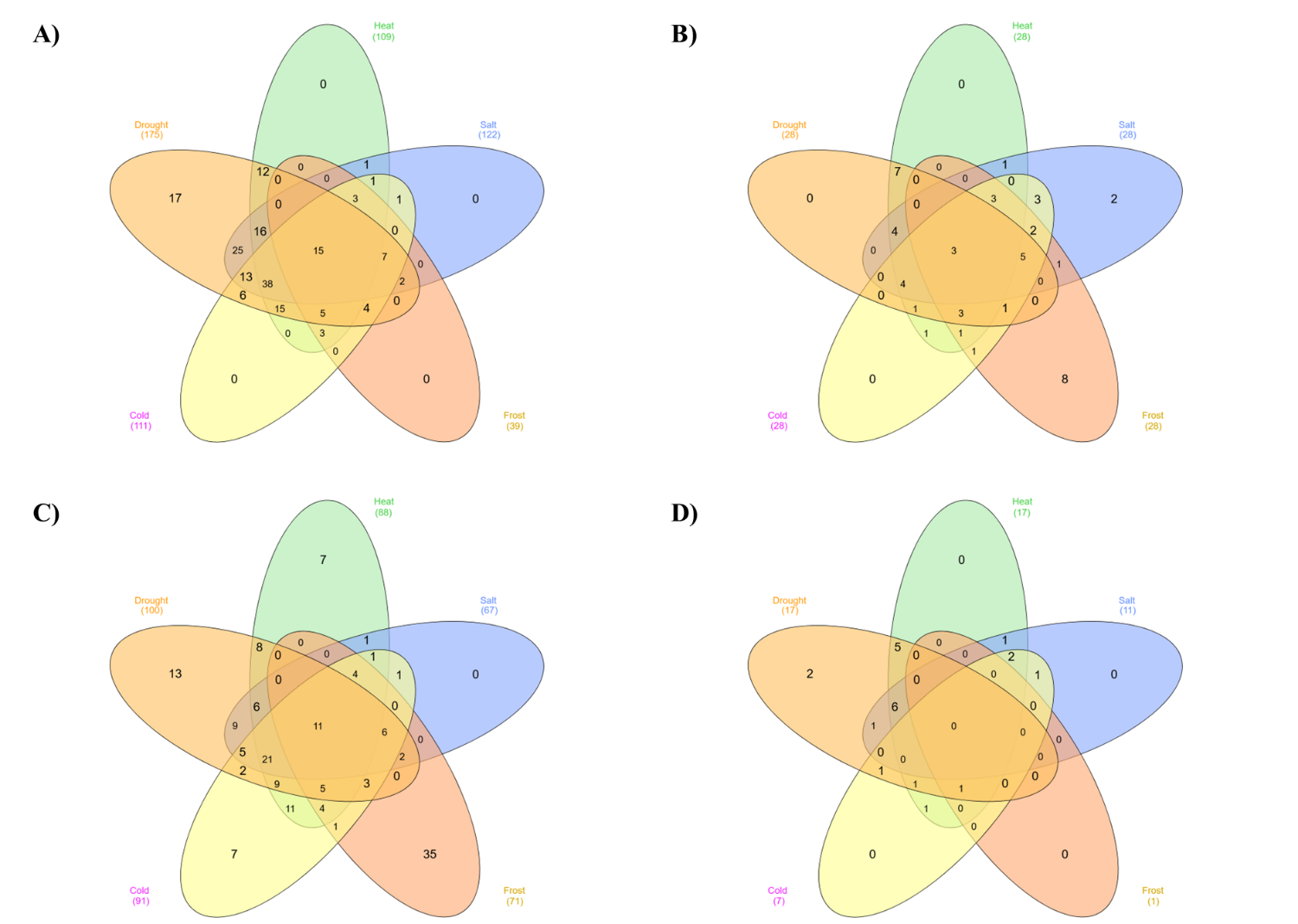
 Figure.** Venn diagram showing the breakdown of the number of genes according to the abiotic stress studied, for each species analyzed. The figure shows broadleaf trees.

(A) *Fagus sylvatica* L., (B) *Quercus pubescens* Willd; (C) *Quercus robur* L., (D) *Quercus ilex* L.

**S2 Figure.** Venn diagram showing the breakdown of the number of genes according to the abiotic stress studied, for each species analyzed. The figure shows coniferus trees.

**
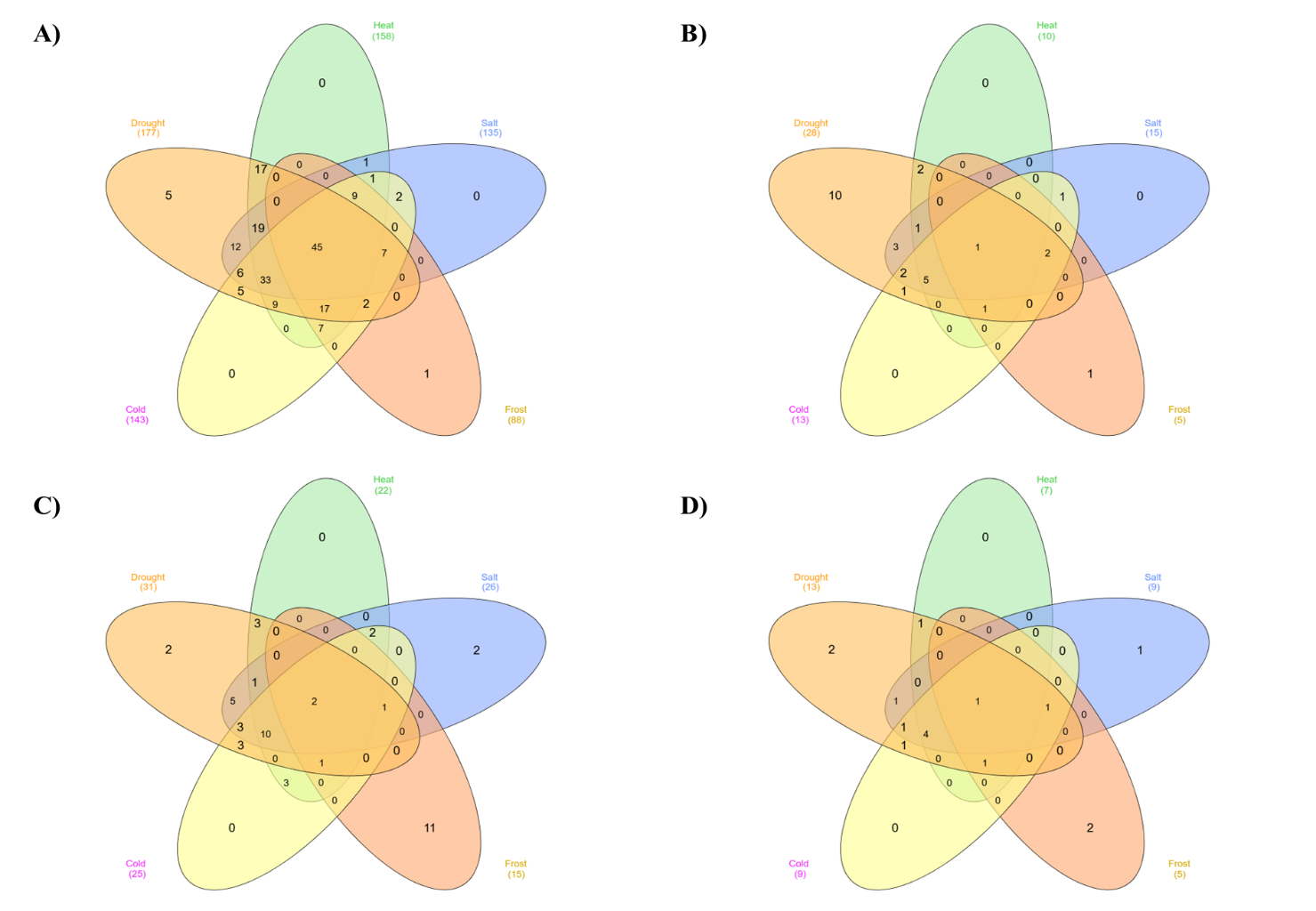
**

(A) *Abies alba* Mill., (B) *Pinus pinea* L.; (C) *Pinus pinaster* Aiton., (D) *Pinus nigra* L.
