# Supplementary material for: Investigating adaptation to environmental variability in forest trees through molecular phylogenetic analysis
Source: PLoS One. 2025 Dec 23;20(12):e0338893. doi: 10.1371/journal.pone.0338893 (PMC12725590; doi:10.1371/journal.pone.0338893)

Investigating adaptation to environmental variability in forest trees through molecular phylogenetic analysis

Cesare Garosi^1#a^, Cristina Vettori^1,2*^, Roberta Ferrante^1,3^, Donatella Paffetti1^1,3^

^1^ Department of Agriculture, Food, Environment and Forestry (DAGRI), University of Florence, Piazzale delle Cascine 18, 50144 Florence, Italy

^2^ National Research Council of Italy (CNR), Institute of Bioscience and Bioresources (IBBR), Division of Florence, Via Madonna del Piano 10, 50019 Sesto Fiorentino, Italy

^3^ NBFC, National Biodiversity Future Center, Palermo 90133, Italy

^#a^Current address: Institute of Research on Terrestrial Ecosystems (IRET), National Research Council of Italy (CNR), Via Madonna del Piano 10, 50019 Sesto Fiorentino, Italy

*** Corresponding author:**

E-mail: [cristina.vettori@cnr.it](mailto:cristina.vettori@cnr.it) (CV)

S2 Appendix: **Values of Substitution rate and graphical representation of substitution per site number in the analysed Phylogenetic groups**

**Table S1.** Substitution rates in conifer protein-coding genes compared to angiosperm genes.

| Pairwise comparison | Gene number | dS (Ks) | d4 (l4) | dN (Ka) | dN/dS (Ka/Ks) |
| --- | --- | --- | --- | --- | --- |
| Gymnosperms: | **265** | 0.4610 | 110.8509 | 0.1976 | 0.4617 |
| *Abies alba* | 196 |  |  |  |  |
| *Pinus pinea* | 28 |  |  |  |  |
| *Pinus pinaster* | 29 |  |  |  |  |
| *Pinus nigra* | 12 |  |  |  |  |
| Angiosperms: | **329** | 1.7439 | 175.4586 | 0.3847 | 0.2206 |
| *Fagus sylvatica* | 180 |  |  |  |  |
| *Quercus robur* | 107 |  |  |  |  |
| *Quercus pubescens* | 28 |  |  |  |  |
| *Quercus ilex* | 14 |  |  |  |  |
| Fold change |  |  |  |  |  |
| Angiosperms/Gymnosperms |  | 3.78:1 | 1.58:1 | 1.94:1 | 1:2.09 |

Mean genetic distances at synonymous (dS), 4-fold degenerate (d4) and non-synonymous (dN) sites are expressed as a number of substitutions per site.

**Figure S1.** Distribution of evolutionary estimates for conifer and angiosperm protein-coding genes. A. Smoothed density plots of dS estimates. B. Smoothed density plot of dN estimates. C. Histogram plots of dN/dS estimates.


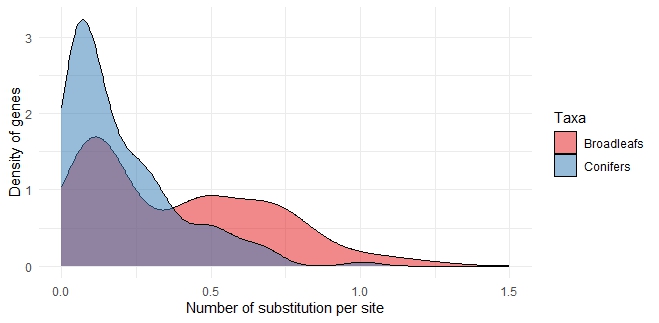

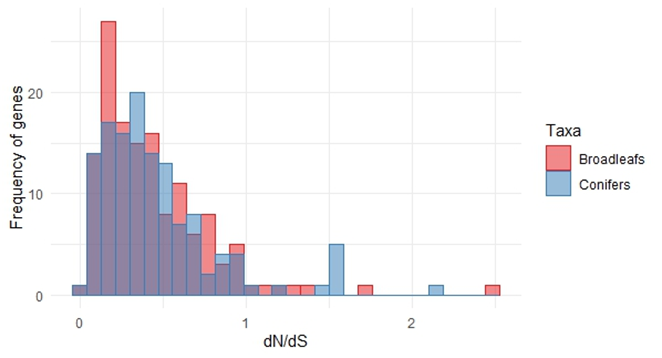

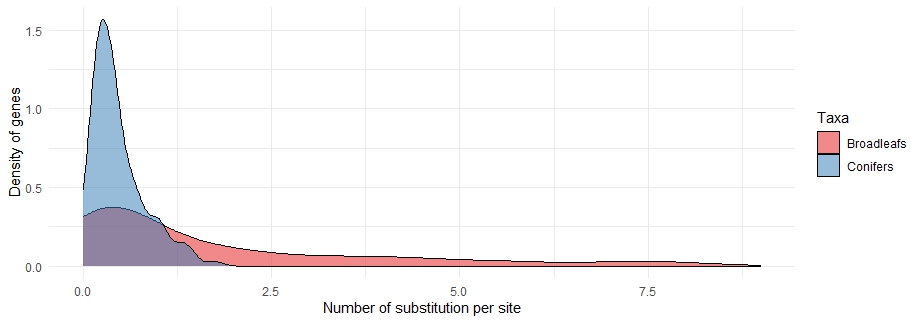


**b)**

**c)**

**a)**

**Table S2.** Substitution rates in conifer protein-coding genes (Drought stress response) compared to angiosperm genes.

| Pairwise comparison | Gene number | Ks (dS) | l4 (d4) | Ka (dN) | Ka/Ks (dN/dS) |
| --- | --- | --- | --- | --- | --- |
| Gymnosperms: | **265** | 0.4760 | 111.1049 | 0.1939 | 0.47144 |
| *Abies alba* | 196 |  |  |  |  |
| *Pinus pinea* | 28 |  |  |  |  |
| *Pinus pinaster* | 29 |  |  |  |  |
| *Pinus nigra* | 12 |  |  |  |  |
| Angiosperms: | **329** | 1.8062 | 172.9264 | 0.3972 | 0.4433 |
| *Fagus sylvatica* | 180 |  |  |  |  |
| *Quercus robur* | 107 |  |  |  |  |
| *Quercus pubescens* | 28 |  |  |  |  |
| *Quercus ilex* | 14 |  |  |  |  |
| Fold change |  |  |  |  |  |
| Angiosperms/Gymnosperms |  | 3.79:1 | 1.06:1 | 2.04:1 | 1:1.06 |

Mean genetic distances at synonymous (dS), 4-fold degenerate (d4) and non-synonymous (dN) sites are expressed as a number of substitutions per site.


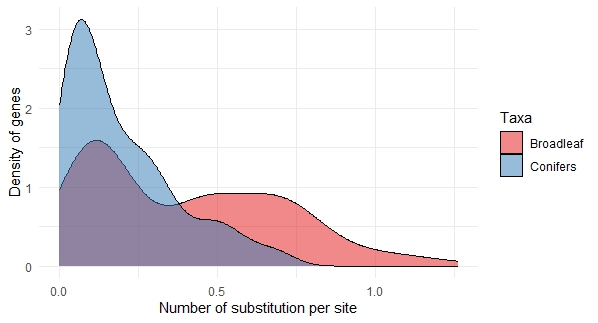
**Figure S2.** Distribution of evolutionary estimates for conifer and angiosperm protein-coding genes (Drought-stress response). A. Smoothed density plots of dS estimates. B. Smoothed density plot of dN estimates. C. Boxplots of dN/dS estimates.

**b)**

**a)**


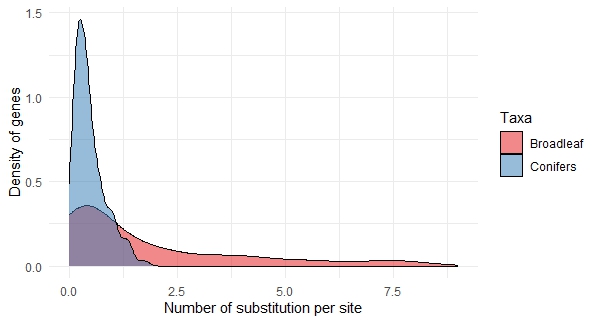


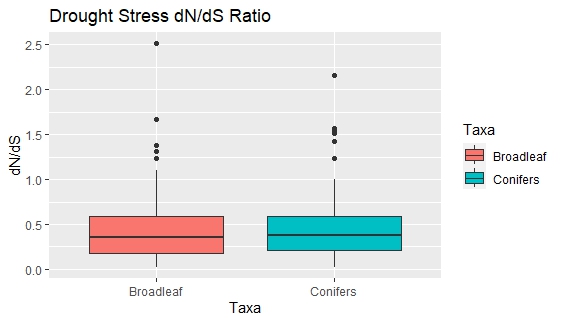


**c)**

**Table S3.** Substitution rates in conifer protein-coding genes (Cold stress response) compared to angiosperm genes.

| Pairwise comparison | Gene number | Ks (dS) | l4 (d4) | Ka (dN) | Ka/Ks (dN/dS) |
| --- | --- | --- | --- | --- | --- |
| Gymnosperms: | **265** | 0.4338 | 113.3603 | 0.2020 | 0.4931 |
| *Abies alba* | 196 |  |  |  |  |
| *Pinus pinea* | 28 |  |  |  |  |
| *Pinus pinaster* | 29 |  |  |  |  |
| *Pinus nigra* | 12 |  |  |  |  |
| Angiosperms: | **329** | 1.7546 | 166.2012 | 0.3799 | 0.4276 |
| *Fagus sylvatica* | 180 |  |  |  |  |
| *Quercus robur* | 107 |  |  |  |  |
| *Quercus pubescens* | 28 |  |  |  |  |
| *Quercus ilex* | 14 |  |  |  |  |
| Fold change |  |  |  |  |  |
| Angiosperms/Gymnosperms |  | 4.05:1 | 1.47:1 | 1.88:1 | 1.15:1 |

Mean genetic distances at synonymous (dS), 4-fold degenerate (d4) and non-synonymous (dN) sites are expressed as a number of substitutions per site.

**Figure S3.** Distribution of evolutionary estimates for conifer and angiosperm protein-coding genes (Cold-stress response). A. Smoothed density plots of dS estimates. B. Smoothed density plot of dN estimates. C. Boxplots of dN/dS estimates.

**a)**

**c)**


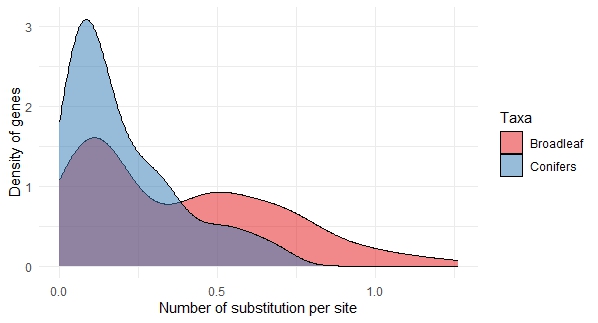

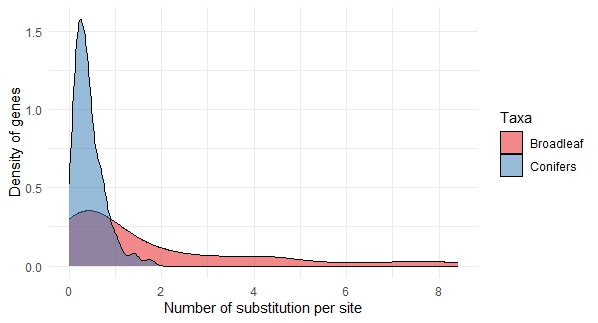

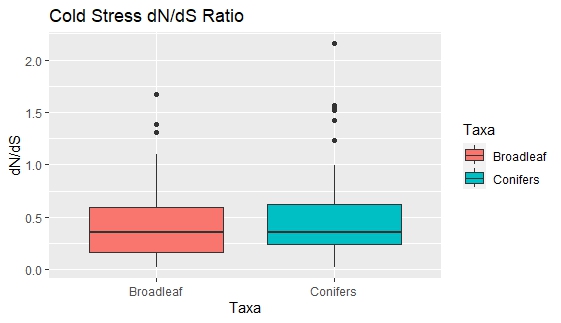


**b)**

**Table S4.** Substitution rates in conifer protein-coding genes (Heat stress response) compared to angiosperm genes.

| Pairwise comparison | Gene number | Ks (dS) | l4 (d4) | Ka (dN) | Ka/Ks (dN/dS) |
| --- | --- | --- | --- | --- | --- |
| Gymnosperms: | **265** | 0.4469 | 122.1841 | 0.2005 | 0.4895 |
| *Abies alba* | 196 |  |  |  |  |
| *Pinus pinea* | 28 |  |  |  |  |
| *Pinus pinaster* | 29 |  |  |  |  |
| *Pinus nigra* | 12 |  |  |  |  |
| Angiosperms: | **329** | 1.7989 | 180.0807 | 0.3863 | 0.4279 |
| *Fagus sylvatica* | 180 |  |  |  |  |
| *Quercus robur* | 107 |  |  |  |  |
| *Quercus pubescens* | 28 |  |  |  |  |
| *Quercus ilex* | 14 |  |  |  |  |
| Fold change |  |  |  |  |  |
| Angiosperms/Gymnosperms |  | 4.03:1 | 1.47:1 | 1.93:1 | 1:1.14 |

Mean genetic distances at synonymous (dS), 4-fold degenerate (d4) and non-synonymous (dN) sites are expressed as a number of substitutions per site.

**Figure S4.** Distribution of evolutionary estimates for conifer and angiosperm protein-coding genes (Heat-stress response). A. Smoothed density plots of dS estimates. B. Smoothed density plot of dN estimates. C. Boxplots of dN/dS estimates.


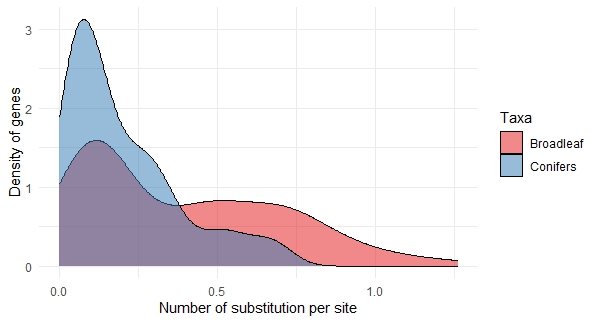


**a)**


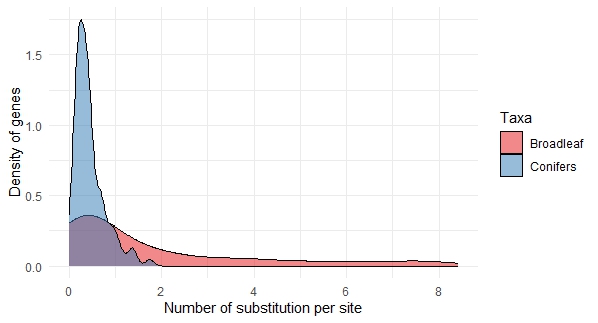


**b)**


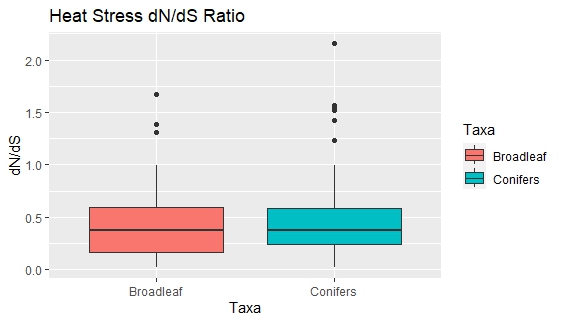


**c)**

**Table S5.** Substitution rates in conifer protein-coding genes (Salt stress response) compared to angiosperm genes.

| Pairwise comparison | Gene number | Ks (dS) | l4 (d4) | Ka (dN) | Ka/Ks (dN/dS) |
| --- | --- | --- | --- | --- | --- |
| Gymnosperms: | **265** | 0.4731 | 106.9135 | 0.1983 | 0.4607 |
| *Abies alba* | 196 |  |  |  |  |
| *Pinus pinea* | 28 |  |  |  |  |
| *Pinus pinaster* | 29 |  |  |  |  |
| *Pinus nigra* | 12 |  |  |  |  |
| Angiosperms: | **329** | 1.7688 | 162.6645 | 0.3959 | 0.4498 |
| *Fagus sylvatica* | 180 |  |  |  |  |
| *Quercus robur* | 107 |  |  |  |  |
| *Quercus pubescens* | 28 |  |  |  |  |
| *Quercus ilex* | 14 |  |  |  |  |
| Fold change |  |  |  |  |  |
| Angiosperms/Gymnosperms |  | 3.74:1 | 1.52:1 | 2.00:1 | 1:1.02 |

Mean genetic distances at synonymous (dS), 4-fold degenerate (d4) and non-synonymous (dN) sites are expressed as a number of substitutions per site.

**Figure S5.** Distribution of evolutionary estimates for conifer and angiosperm protein-coding genes (Salt-stress response). A. Smoothed density plots of dS estimates. B. Smoothed density plot of dN estimates. C. Boxplots of dN/dS estimates.


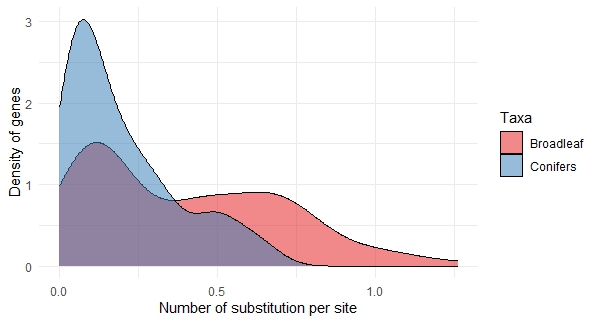


**a)**


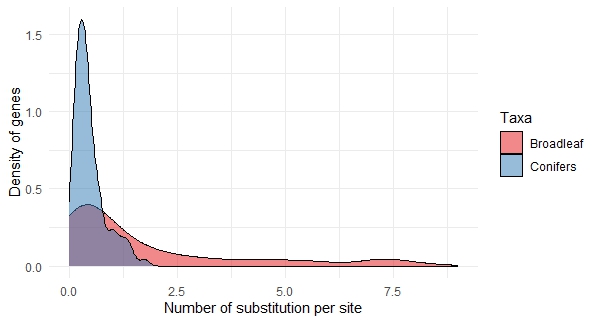


**b)**


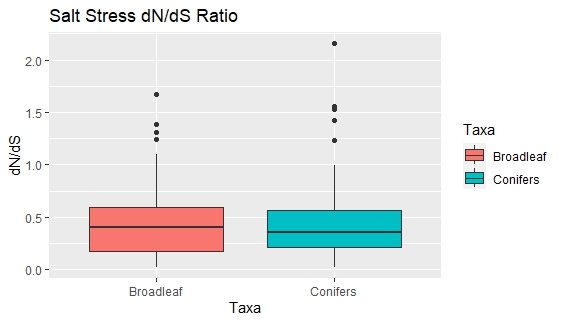


**c)**

**Table S6.** Substitution rates in conifer protein-coding genes (Frost stress response) compared to angiosperm genes.

| Pairwise comparison | Gene number | Ks (dS) | l4 (d4) | Ka (dN) | Ka/Ks (dN/dS) |
| --- | --- | --- | --- | --- | --- |
| Gymnosperms: | **265** | 0.4299 | 120.4651 | 0.1997 | 0.4753 |
| *Abies alba* | 196 |  |  |  |  |
| *Pinus pinea* | 28 |  |  |  |  |
| *Pinus pinaster* | 29 |  |  |  |  |
| *Pinus nigra* | 12 |  |  |  |  |
| Angiosperms: | **329** | 1.9465 | 164.6913 | 0.3973 | 0.3665 |
| *Fagus sylvatica* | 180 |  |  |  |  |
| *Quercus robur* | 107 |  |  |  |  |
| *Quercus pubescens* | 28 |  |  |  |  |
| *Quercus ilex* | 14 |  |  |  |  |
| Fold change |  |  |  |  |  |
| Angiosperms/Gymnosperms |  | 4.53:1 | 1.37:1 | 1.99:1 | 1:1.30 |

Mean genetic distances at synonymous (dS), 4-fold degenerate (d4) and non-synonymous (dN) sites are expressed as a number of substitutions per site.

**Figure S6.** Distribution of evolutionary estimates for conifer and angiosperm protein-coding genes (Frost-stress response). A. Smoothed density plots of dS estimates. B. Smoothed density plot of dN estimates. C. Boxplots of dN/dS estimates.


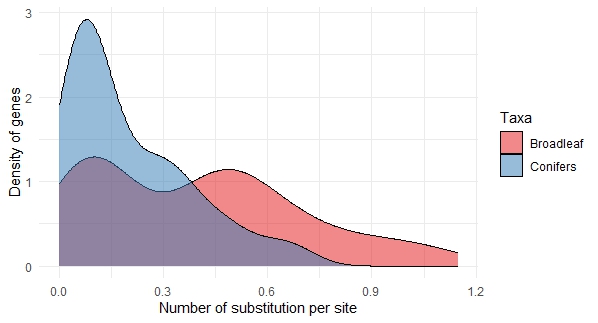


**a)**


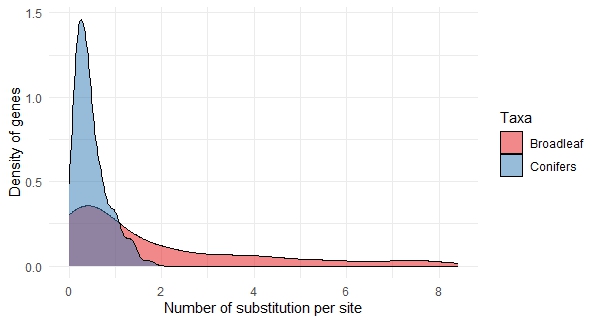


**c)**

**b)**


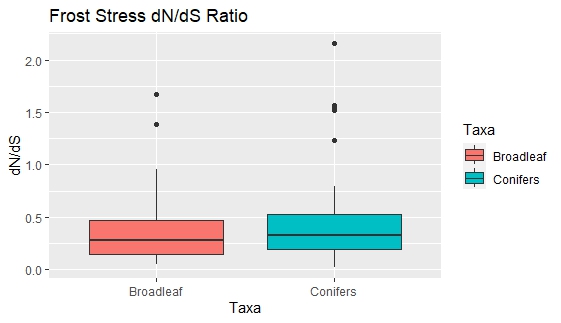

Supplement: S2 File — Values of Substitution rate and graphical representation of substitution per site number in the analysed Phylogenetic groups. (DOCX) [file pone.0338893.s011.docx]
